# Consortia of low-abundance bacteria drive sulfate reduction-dependent degradation of fermentation products in peat soil microcosms

**DOI:** 10.1038/ismej.2016.42

**Published:** 2016-03-25

**Authors:** Bela Hausmann, Klaus-Holger Knorr, Katharina Schreck, Susannah G Tringe, Tijana Glavina del Rio, Alexander Loy, Michael Pester

**Affiliations:** 1 Division of Microbial Ecology, Department of Microbiology and Ecosystem Science, Research Network Chemistry Meets Microbiology, University of Vienna, Vienna, Austria; 2 Department of Biology, University of Konstanz, Konstanz, Germany; 3 Hydrology Group, Institute of Landscape Ecology, University of Münster, Münster, Germany; 4 Joint Genome Institute, US Department of Energy, Walnut Creek, CA, USA

## Abstract

Dissimilatory sulfate reduction in peatlands is sustained by a cryptic sulfur cycle and effectively competes with methanogenic degradation pathways. In a series of peat soil microcosms incubated over 50 days, we identified bacterial consortia that responded to small, periodic additions of individual fermentation products (formate, acetate, propionate, lactate or butyrate) in the presence or absence of sulfate. Under sulfate supplementation, net sulfate turnover (ST) steadily increased to 16–174 nmol cm^–3^ per day and almost completely blocked methanogenesis. 16S rRNA gene and cDNA amplicon sequencing identified microorganisms whose increases in ribosome numbers strongly correlated to ST. Natively abundant (⩾0.1% estimated genome abundance) species-level operational taxonomic units (OTUs) showed no significant response to sulfate. In contrast, low-abundance OTUs responded significantly to sulfate in incubations with propionate, lactate and butyrate. These OTUs included members of recognized sulfate-reducing taxa (*Desulfosporosinus*, *Desulfopila*, *Desulfomonile*, *Desulfovibrio*) and also members of taxa that are either yet unknown sulfate reducers or metabolic interaction partners thereof. Most responsive OTUs markedly increased their ribosome content but only weakly increased in abundance. Responsive *Desulfosporosinus* OTUs even maintained a constantly low population size throughout 50 days, which suggests a novel strategy of rare biosphere members to display activity. Interestingly, two OTUs of the non-sulfate-reducing genus *Telmatospirillum* (*Alphaproteobacteria*) showed strongly contrasting preferences towards sulfate in butyrate-amended microcosms, corroborating that closely related microorganisms are not necessarily ecologically coherent. We show that diverse consortia of low-abundance microorganisms can perform peat soil sulfate reduction, a process that exerts control on methane production in these climate-relevant ecosystems.

## Introduction

Peatlands currently store about one-third of all terrestrial carbon (Limpens *et al.*, 2008), which is predicted to be partially released as the greenhouse gases CO_2_ and CH_4_ because of climate change (Hodgkins *et al.*, 2014; Schuur *et al.*, 2015). In these water-saturated soils, steep gradients in redox conditions sustain a complex network of biogeochemical processes (Limpens *et al.*, 2008), with organic matter degradation being carried out by different functional guilds of microorganisms. This diversity of simultaneously occurring organic carbon degradation pathways is favored by frequently fluctuating concentrations of electron acceptors (Schmalenberger *et al.*, 2007; Küsel *et al.*, 2008; Knorr and Blodau, 2009) and donors (Schmalenberger *et al.*, 2007; Küsel *et al.*, 2008; Wüst *et al.*, 2009). A changing water table that steadily shifts the oxic–anoxic interface (Knorr *et al.*, 2009; Reiche *et al.*, 2009) and complex flow paths of infiltrating and exfiltrating water create distinct spatial and temporal patterns (hot spots and hot moments) of various biogeochemical processes, making peatlands very dynamic ecosystems (Jacks and Norrström, 2004; Knorr *et al.*, 2009; Knorr and Blodau, 2009; Frei *et al.*, 2012).

Below the water table, where anoxic conditions persist, the processes of fermentation and subsequent methanogenesis are competing with energetically more favorable degradation pathways such as anoxic respiration coupled to nitrate, Fe(III), humic matter or sulfate reduction (Loy *et al.*, 2004; Beer *et al.*, 2008; Hamberger *et al.*, 2008; Küsel *et al.*, 2008; Drake *et al.*, 2009; Keller *et al.*, 2009; Knorr and Blodau, 2009; Knorr *et al.*, 2009; Reiche *et al.*, 2009; Wüst *et al.*, 2009; Hunger *et al.*, 2011; Pester *et al.*, 2012b). The importance of sulfate reduction for peatland biogeochemistry is often neglected because of the low prevailing sulfate concentrations (μm range). This is contrasted by a highly active but cryptic sulfur cycle that rapidly reoxidizes reduced sulfur species and thus sustains sulfate reduction rates that are as high as in sulfate-rich marine surface sediments (Pester *et al.*, 2012b). Here, (bio)geochemical sulfur reoxidation can be either coupled to the reduction of O_2_ at the oxic–anoxic interphase or to the reduction of Fe(III) (Hansel *et al.*, 2015) and/or humic matter (Heitmann and Blodau, 2006; Yu *et al.*, 2015) under completely anoxic conditions. As sulfate-reducing microorganisms (SRM) generally outcompete methanogens and syntrophically associated fermenters (Muyzer and Stams, 2008), they exert an important intrinsic control function on CH_4_ production in peatlands (Gauci *et al.*, 2004, 2005; Gauci and Chapman, 2006).

Microbial communities in soils are typically composed of a large number of species (e.g., Roesch *et al.*, 2007; Serkebaeva *et al.*, 2013). Few of these taxa are very or moderately abundant (arbitrarily classified at ⩾1% and ⩾0.1% relative abundance, respectively). The remaining majority of species have an individual relative abundance of <0.1% and are summarized as the rare biosphere (Pedrós-Alió, 2012; Lynch and Neufeld, 2015). Because of its species richness and genetic diversity, the rare biosphere functions as a microbial seed bank for the recruitment of dormant cells to become active and numerically dominant under favorable environmental conditions. For example, singular disturbance events such as oil spills resulted either in the recruitment of specific taxa (Teira *et al.*, 2007) or even widespread changes of the total microbial community (Newton *et al.*, 2013). Furthermore, periodic environmental filtering through seasonal changes led to reoccurring temporal shifts between rare and abundant population sizes in marine bacterioplankton species (Campbell *et al.*, 2011; Vergin *et al.*, 2013; Alonso-Sáez *et al.*, 2015). Dormant microorganisms may also be introduced to environments by transport mechanisms such as ocean currents (Müller *et al.*, 2014) or wind (Hervàs and Casamayor, 2009; Hervàs *et al.*, 2009), where they stay inactive and eventually undergo taphonomy (Lynch and Neufeld, 2015). However, there is increasing evidence that the rare biosphere is not just a seed bank but also harbors active populations. Seasonal patterns in marine bacterial and archaeal plankton revealed that many taxa that displayed reoccurring annual abundance changes were actually rare biosphere members and even during their bloom periods never reached abundant numbers (Campbell *et al.*, 2011; Hugoni *et al.*, 2013; Alonso-Sáez *et al.*, 2015). In addition, microorganisms with a specialized metabolism often fulfill gatekeeper functions in an ecosystem while sustaining low-abundance populations (Lynch and Neufeld, 2015), for example, N_2_-fixing microorganisms in the ocean (Großkopf *et al.*, 2012) or nitrifiers in wastewater treatment plants (Wang *et al.*, 2014). The reasons for their constantly low abundance are not yet resolved but most likely vary between different taxa and may include a decreased efficiency in the competition for resources, predation and viral attack, or an energy metabolism sustaining little growth (Lynch and Neufeld, 2015).

Recognized SRM are typically members of the rare biosphere in peatlands (Loy *et al.*, 2004; Costello and Schmidt, 2006; Dedysh *et al.*, 2006; Kraigher *et al.*, 2006; Pester *et al.*, 2010; Steger *et al.*, 2011; Tveit *et al.*, 2013). Previous research showed that rare peatland *Desulfosporosinus* spp. have the potential for high cell-specific sulfate reduction rates (Pester *et al.*, 2010) that are at the upper limit of cell-specific rates observed for pure cultures (Detmers *et al.*, 2001). This provided indirect evidence that rare biosphere members could contribute substantially to biogeochemical cycling in peatlands. In the same peatland, a high diversity of deep-branching lineages of the *dsrAB* genes (subunit A and B of the dissimilatory sulfite reductase) was identified previously (Loy *et al.*, 2004; Schmalenberger *et al.*, 2007; Pester *et al.*, 2010; Steger *et al.*, 2011). Although *dsrAB* generally serve as functional marker genes for SRM (Müller *et al.*, 2015), the taxonomic identity of these novel lineages and their function with regard to sulfur and carbon metabolism remain unclear (Pester *et al.*, 2012b; Müller *et al.*, 2015). Here, we set up controlled incubation experiments supplemented with single substrates and sulfate to simulate sulfate-reducing hot spots and gain insights into the discrepancy between the low abundance of SRM and the high sulfate reduction rate regularly observed in peatlands.

## Materials and methods

### Peat microcosms

The acidic peatland Schlöppnerbrunnen II is located in Germany (50°07′54.8″N, 11°52′51.8″E). Main site characteristics and soil core sampling are described in the Supplementary Methods. Peat soil microcosms were set up by mixing 30 g of fresh soil (10–20 cm depth) with 60 ml of filter-sterilized (0.2 μm) and anoxic peat water in 250 ml sterile glass bottles. Microcosms were sealed under 100% N_2_ with butyl rubber septa and incubated for 53 days in the dark at 14 °C, which is within the observed air temperature range of this peatland (Schmidt *et al.*, 2015). Microcosms were periodically amended from the first day of incubation with formate, acetate, propionate, lactate or butyrate, or incubated without any external substrate (Supplementary Figure S1). Added substrates generally did not exceed a final concentration of 100–200 μm (Supplementary Figure S2a). A side aspect of the formate additions was its potential to target H_2_-using microorganisms as well. Interconversion of formate to H_2_+CO_2_ is at thermodynamic equilibrium (−4 kJ mol^–1^) and has been documented in peat soil incubations of the analyzed peatland (Hunger *et al.*, 2011). Half of the substrate-amended and -unamended microcosms additionally received periodic amendments of sulfate (Supplementary Figure S1). These microcosms were initially spiked with sulfate to a final concentration of 190–387 μm, and, thereafter, were periodically amended with small amounts of sulfate equivalent to 79–161 μm (Supplementary Figure S2b). Triplicate microcosms were set up per incubation condition (36 microcosms in total) and periodically sampled for substrate/sulfate concentrations directly after amendment (Supplementary Figure S1). Identical microcosms were set up to measure CO_2_ and CH_4_ production (Supplementary Methods).

### Quantification of sulfate and substrate turnover

Sulfate and substrate concentrations at days 0, 4, 7, 11, 14, 18, 21, 25, 28, 35, 42 and 49 were determined by capillary electrophoresis (P/ACE MDQ; Beckman Coulter, Brea, CA, USA) directly after amendment using the CEofix Anions 5 Kit (Analis, Suarlée, Belgium). To obtain substrate-specific sulfate turnover (ST) rates, we determined how much sulfate was used in the substrate- and sulfate-amended microcosms in comparison with the sulfate-amended controls without external substrate. This served to discriminate against sulfate dissimilation caused by SRMs using endogenous substrates and sulfate assimilation in general. The calculation was performed by subtracting average sulfate concentrations of the sulfate-amended no-substrate controls from the individual substrate- and sulfate-amended microcosms at each corresponding time point. A nonparametric regression was fitted to the obtained values in R (R Core Team, 2015), and the slope of the regression curve was used to calculate net ST rates. Sulfate concentrations were previously converted from μm to nmol cm^–3^ of fresh peat soil using the measured soil water content (78% Supplementary Methods) and the bulk density of peat soil (0.29 g cm^–3^; Goldberg *et al.*, 2008) to allow comparison with results obtained in other studies (e.g., Knorr *et al.*, 2009; Knorr and Blodau, 2009).

### Amplicon sequencing and qPCR

After 5, 26 and 50 days of incubation, total nucleic acids were extracted from soil samples, purified, separated into RNA and DNA fractions and quantified (Supplementary Methods). Complete removal of DNA in RNA samples was verified with a quantitative PCR (qPCR) assay targeting the 16S rRNA genes of most *Bacteria* and *Archaea* (Supplementary Methods). The V4 region of the 16S rRNA gene and its cDNA was amplified as described previously (Caporaso *et al.*, 2011) and sequenced in three Illumina MiSeq runs at the Joint Genome Institute (genome portal projects 1 016 201, 1 016 203 and 1 031 338) and deposited at NCBI (SRA277144, SRA277986 and SRA277988). All reads were subjected to quality control (iTagger; https://bitbucket.org/berkeleylab/jgi_itagger/), UPARSE clustering (including *de novo* chimera filtering) (Edgar, 2013), singleton filtering (iTagger) and UCHIME chimera filtering (Edgar *et al.*, 2011) (Supplementary Methods). This resulted in 10.0 and 11.3 million high-quality reads from DNA and RNA samples, respectively, which formed a total of 7435 species-level operational taxonomic units (OTUs) (97% sequence identity). The library size varied between 17 795–323 368 and 46 764–341 948 reads per DNA and RNA sample, respectively, with a median of 84 629 and 93 245 reads (Supplementary Figure S3a). Taxonomic identity was assigned with the Ribosomal Database Project (RDP) classifier 2.9 and the RDP 16S rRNA training set 10 using a confidence threshold of 0.5 (Wang *et al.*, 2007). Relative abundances obtained from 16S rRNA gene samples were corrected for copy number bias using *rrn*DB 4.3.3 (Stoddard *et al.*, 2015) in R (R Core Team, 2015), resulting in estimated relative genome abundance values for each OTU (Supplementary Methods). This was previously shown to improve diversity and abundance estimates (Kembel *et al.*, 2012). For selected OTUs, the taxonomic assignment was verified by phylogenetic tree reconstruction as outlined in the Supplementary Methods. qPCR assays targeting 16S rRNA genes or cDNA of the genus *Desulfosporosinus*, most *Bacteria* and *Archaea*, and selected *dsrA* variants were performed as described in the Supplementary Methods. Reliability of relative abundance shifts in our amplicon sequencing approach was verified by a mock community analysis as internal control (Supplementary Figure S4; Herbold *et al.*, 2015).

### Statistical analysis

**Figure 1:**
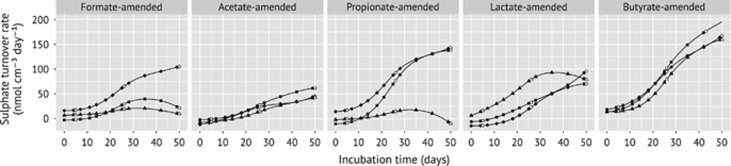
Substrate-specific net ST rates in sulfate-stimulated microcosm. Individual microcosms are depicted by different symbols (and are consistent in all figures that show triplicate data). Solid symbols indicate time points where sulfate concentrations were measured, and open symbols indicate time points selected for 16S rRNA (gene) amplicon sequencing. Minor negative values are because of variations caused by manual sulfate addition to individual microcosms in comparison with the corresponding no-substrate controls and as such can be neglected. Sulfate pools were never depleted in any sulfate-amended microcosm.

Differential abundance of OTUs between individual treatments and time points was tested in R (R Core Team, 2015) using the package edgeR (Robinson *et al.*, 2010) based on the recommendations of McMurdie and Holmes (2014). Pairwise comparisons were performed by testing independently for the effect of sulfate, substrate and incubation time (Supplementary Methods). Each test included three replicates per treatment, with the exception of the propionate- and sulfate-amended microcosms, where one replicate was excluded from all analyses because of its inconsistent ST (Figure 1). OTUs entering statistical analysis required >10 reads in at least two out of six samples per pairwise comparison. Significantly responding OTUs were assigned to sulfate-stimulated or -deterred conditions with respect to a given substrate and response time. For association network analysis, regularized log transformation was applied to OTU abundance data to stabilize variance heterogeneity, as implemented in the DESeq2 package (Love *et al.*, 2014). Resulting variance homogeneity was verified with the Brown–Forsythe test (*P*-value>0.1) in R (R Core Team, 2015). Transformed OTU abundances of responsive OTUs were correlated pairwise to each other and to ST using Pearson's correlation. Significant (false discovery rate-corrected *P*-value<0.05) OTU–ST and OTU–OTU pairwise correlations were used for network construction.

### Physiological characterization of *Telmatospirillum siberiense*

*Telmatospirillum siberiense* 26-4b1^T^ (DSM-18240; Sizova *et al.*, 2007) was incubated anoxically with DSMZ medium 1126 (DSMZ, Braunschweig, Germany). Sulfate was added to the medium to a final concentration of 1 mm. Anaerobic growth at 28 °C was inferred from measuring optical density (600 nm) in parallel incubations in medium supplemented with different carbon sources, that is, citrate (positive control for growth of *T. siberiense*), formate, propionate, lactate or butyrate (5 mm each). DNA was extracted from biomass grown on the citrate-amended medium with the DNeasy Blood and Tissue Kit (Qiagen, Hilden, Germany) and tested for the presence of the *dsrAB* genes with the primer pairs DSR190F/DSR916R (*dsrA*), DSR1762F/DSR2107R (*dsrB*) and DSR190F/DSR2107R (both) (Pelikan *et al.*, 2015).

## Results

### Fermentation products differ in their impact on ST

Anoxic peat microcosms were set up to reflect naturally occurring concentrations of substrates and generally did not exceed 100–200 μm (Supplementary Figure S2a). For this purpose, microcosms were periodically amended with small amounts of formate, acetate, propionate, lactate or butyrate—substrates that represent typical organic carbon degradation intermediates in peatlands (Schmalenberger *et al.*, 2007; Küsel *et al.*, 2008; Limpens *et al.*, 2008). Microcosms without external substrate served as controls for the impact of endogenous substrates. To mimic a sulfate reduction hot spot in the peat (Jacks and Norrström, 2004; Knorr and Blodau, 2009; Knorr *et al.*, 2009; Frei *et al.*, 2012), half of the microcosms were periodically amended with sulfate in the lower μm range (accumulating over time to a maximum of ~0.5 to 2 mm depending on the substrate supplemented in parallel; Supplementary Figure S2b). In sulfate-stimulated incubations, supplemented substrates were completely turned over after each addition. The same was true for incubations without external sulfate with the exception of butyrate-amended microcosms, where butyrate started to accumulate slightly after 30 days (Supplementary Figure S2a).

Net ST increased over time but differed substantially between different substrates. Highest ST was observed for butyrate at the end of the incubations (averaging 174 nmol cm^–3^ per day), followed by propionate, lactate, acetate and formate (averaging 16 nmol cm^–3^ per day) in decreasing order (Figure 1). In formate- and propionate-amended microcosms, one replicate each showed a clear deviation in ST from the other two, being either substantially higher or lower, respectively. As the peat soil had not been homogenized before separation into individual microcosms, this likely reflects the spatially heterogeneous distribution of the microbiota in the peat soil matrix. ST increased overincubation time in each of the individual substrate-amended microcosms. Little ST at early time points is possibly due to a presence of alternative, energetically more favorable electron acceptors such as nitrate, Fe(III) or humic matter (Thauer *et al.*, 1977) as observed previously in this peatland (Küsel *et al.*, 2008; Knorr and Blodau, 2009). The presence of such alternative electron acceptors was corroborated by a parallel microcosm setup, where over the total incubation time of 27 days only a minor carbon flow towards methanogenesis (0.1–0.2%) was observed in incubations not amended with sulfate (Supplementary Table S1). In sulfate-stimulated microcosms, CH_4_ production was reduced by 83–100% as compared with controls without additional sulfate (based on the time frame between 18 and 27 days; Supplementary Figure S2c). In the same parallel microcosm setup, sulfate amendment did not alter total CO_2_ production (Supplementary Figure S2c), but shifted 43–100% of organic carbon mineralization towards sulfate reduction at the very end of the incubation time (assuming that equivalents of acetate are completely oxidized to two CO_2_ with the eight released reducing equivalents being used to reduce sulfate to sulfide, detailed in Supplementary Methods and Results).

### Only low-abundance OTUs responded to sulfate stimulation

**Figure 2:**
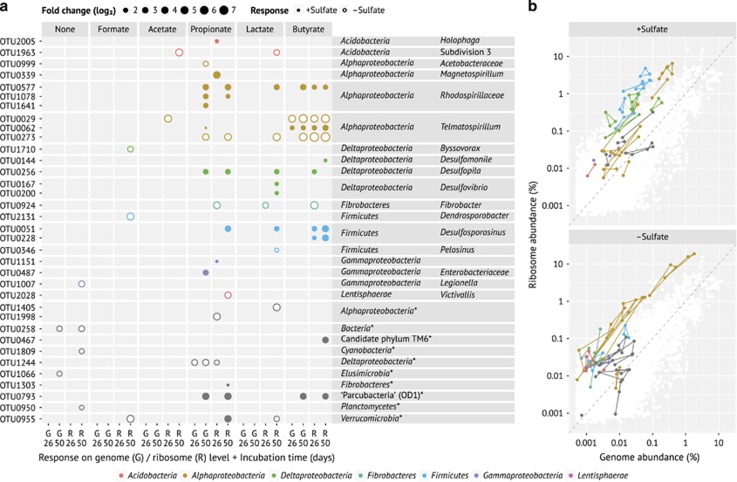
OTUs responding to individual carbon degradation intermediates. (**a**) OTUs responding positively to sulfate stimulation (+Sulfate) and/or unamended conditions (−Sulfate) at the 16S rRNA cDNA (i.e., ribosome) or gene (i.e., genome) level. OTUs 0062+0273, 0346 or 0029+0062 exhibited both +Sulfate and −Sulfate responses in propionate-, lactate- or butyrate-amended microcosms, respectively (stronger response indicated). Taxonomic classification indicates phyla (classes for *Proteobacteria*) and the highest taxonomic level resolved by the RDP classifier. OTUs without RDP classification at class level (marked with asterisks) were assigned tentative classifications by the SINA online classifier (Pruesse *et al.*, 2012) using a minimum identity threshold of 0.75. (**b**) Relationship of relative genome and ribosome abundance per responsive OTU at time points with significant abundance shifts. Lines connect replicate microcosms for each OTU (abundances of zero not shown). White circles represent 10 000 randomly selected data points (>0.0005% relative abundance).

We used changes in relative genome abundance (i.e., relative 16S rRNA gene abundance corrected for *rrn* operon copy numbers) per OTU to estimate microbial growth. In addition, the protein synthesis potential of an OTU population was determined by calculating its relative ribosome abundance (i.e., relative 16S rRNA cDNA abundance). The abundant community (OTUs with ⩾0.1% relative genome abundance) of the native soil was dominated by members of the *Acidobacteria*, *Alphaproteobacteria*, *Actinobacteria* and *Planctomycetes* (Supplementary Figure S3b). None of these abundant OTUs was stimulated by sulfate in any of the treatments. In contrast, 18 low-abundance OTUs each responded positively (*P*-value<0.05) to either sulfate-stimulated or -unstimulated conditions under different substrate scenarios (Figure 2a). Most of these OTUs stayed below or reached levels slightly above 0.1% relative genome abundance throughout the incubation period. Only *Telmatospirillum* OTU0029 exceeded 1% relative genome abundance towards the end of the incubation period in butyrate incubations without sulfate amendments (Figure 2b).

**Figure 3:**
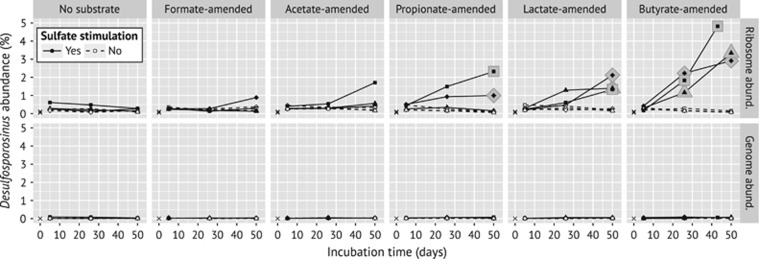
Temporal changes in relative ribosome and genome abundance of the most abundant *Desulfosporosinus* OTU (0051) in incubations with different substrates. Solid lines and symbols depict sulfate-stimulated microcosms, whereas dashed lines and open symbols depict controls without additional sulfate. Diagonal crosses depict the abundance in the native soil, which was sampled from parallel peat soil subsamples and plotted at day 0 in all panels. Halos around symbols indicate significantly higher abundance in the sulfate-stimulated microcosms as compared with their respective controls and day 5.

In accordance to the high ST observed under butyrate, propionate and lactate, 5–11 OTUs responded positively to sulfate stimulation under each of these substrates, whereas none responded significantly to the addition of formate or acetate. Responsive OTUs were affiliated with taxa containing known SRM (*Desulfosporosinus*, *Desulfomonile*, *Desulfopila* and *Desulfovibrio*) and also to taxa not known to harbor SRM. The latter were affiliated to *Alphaproteobacteria*, *Acidobacteria* and *Gammaproteobacteria*, and tentatively also to candidate phylum TM6, *Fibrobacteres*, ‘Parcubacteria' (candidate phylum OD1; Rinke *et al.*, 2013) and *Verrucomicrobia* (Figure 2a). Our stringent statistical approach did not reveal any OTUs responding positively to sulfate stimulation with formate. However, although ST only increased minimally in two of the formate-amended microcosms, it clearly increased in the third microcosm (Figure 1). Interestingly, these differences in ST were mirrored in concurrent response patterns at the ribosome level for several OTUs that responded also positively to the sulfate addition under other substrates (*Desulfosporosinus* OTU0051, *Telmatospirillum* OTU0062, *Desulfomonile* OTU0144, *Desulfopila* OTU0256, *Magnetospirillum* OTU0339 and unclassified *Rhodospirillaceae* OTU0577; Figure 3 and Supplementary Figures S5a–e).

### Low-abundance bacteria differ in their response strategy towards favorable conditions

The majority of OTUs with significantly increased ribosome abundance showed only a weak and some even no significant increase in genome abundance over a period of 50 days (Figure 2a). These relative abundance shifts were not obscured by changes in absolute microbial abundance as the number of bacterial and archaeal 16S rRNA genes cm^–3^ soil remained constant throughout all incubations as measured by qPCR (Supplementary Figure S6a). Members of the genus *Desulfosporosinus* (Supplementary Figure S7a) were major responders to the addition of sulfate and substrate at the ribosome level but apparently did not grow. Two *Desulfosporosinus* OTUs were detected with a relative genome abundance of 0.003% (OTU0051) and 0.001% (OTU0228) in the native soil. Their relative genome abundance was slightly higher at the first measured time point in the microcosms (day 5, on average 0.022% and 0.009%, respectively), which could simply be due to the different soil subsamples used to characterize the native peat soil community and for setting up the microcosms. Alternatively, this might be explained by germination of a *Desulfosporosinus* sub-population (Stackebrandt, 2014), as the nucleic acids extraction from spores is less efficient than from active cells. Thereafter, the relative genome abundance of both OTUs did not change significantly over 50 days of incubation, as confirmed with a qPCR assay targeting the genus *Desulfosporosinus* (Supplementary Figure S6b). Also here, no growth was observed at a median abundance of 1.2 × 10^5^ genomes cm^–3^ soil with one small outlier under lactate at day 50 that reached an abundance of 6.9 × 10^5^ genomes cm^–3^ soil. In contrast, the relative ribosome abundance of both OTUs increased significantly (*P*-value<0.001) from 0.30% to 3.69% (OTU0051) and 0.03% to 0.48% (OTU0228) between days 5 and 50 in microcosms with butyrate and sulfate amendment (Figure 3 and Supplementary Figure S5f). The more abundant *Desulfosporosinus* OTU0051 also responded in the same manner to additions of lactate and propionate. These findings were corroborated by qPCR analysis, which showed that the ribosome per genome ratio of the genus *Desulfosporosinus* increased from 2600–7300 to 57 000–84 000 throughout the incubation in sulfate-stimulated microcosms amended with propionate, lactate or butyrate (Supplementary Figure S6b). Such high cellular ribosome contents have been reported for exponentially growing pure cultures (Bremer and Dennis, 1996) but so far have not been observed in low-abundance microorganisms keeping a steady population size. Among recognized SRM OTUs, *Desulfosporosinus* OTU0051 correlated best with substrate-specific ST (Supplementary Figure S8) and dominated the average relative ribosome abundance of this metabolic group (Figure 2b).

**Figure 4:**
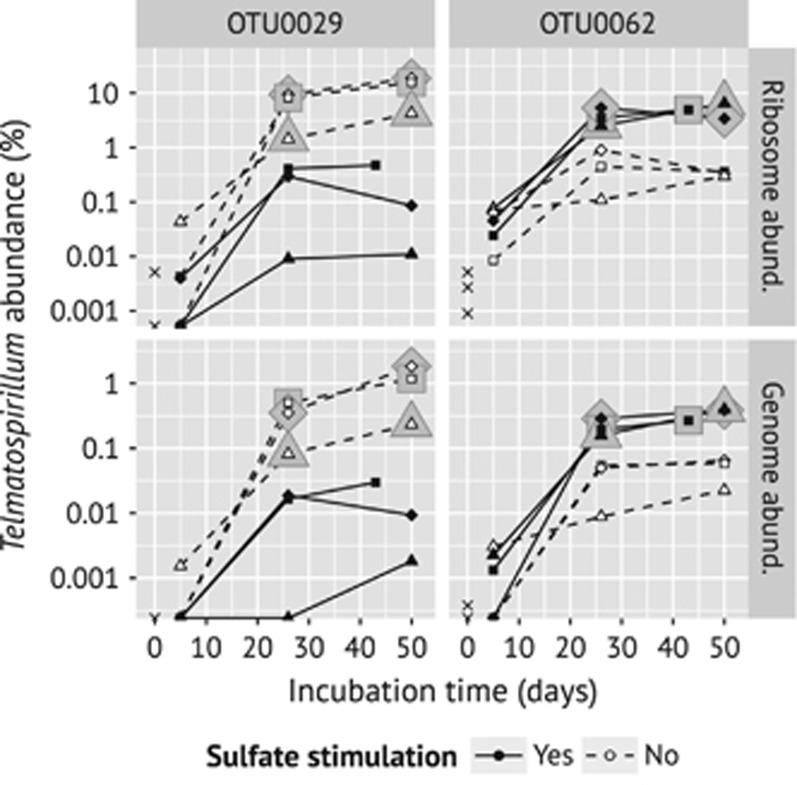
Temporal changes in relative ribosome and genome abundance of the two most abundant *Telmatospirillum* OTUs (0029 and 0062) in butyrate-amended incubations. Solid lines and symbols depict sulfate-stimulated microcosms, whereas dashed lines and open symbols depict controls without additional sulfate (note the logarithmic scale on the y axis). Diagonal crosses depict the abundance in the native soil. Halos around symbols indicate significantly higher abundance in the unstimulated or sulfate-stimulated microcosms, respectively, as compared with their respective controls and day 5. Both *Telmatospirillum* OTUs showed a significant increase in relative ribosome abundance over time, irrespective of sulfate stimulation. Data points drawn directly on the x axis represent relative abundance values of zero.

In contrast to *Desulfosporosinus* and other slow- or non-growing taxa, OTUs of the genus *Telmatospirillum* (Supplementary Figure S7b) responded with both increased relative ribosome and genome abundance (Supplementary Figure S5a). The two most abundant *Telmatospirillum* OTUs were standing out among the responsive OTUs that are not affiliated with recognized SRM because of the magnitude of their response as well as their ecologically incoherent behavior, that is, they showed an opposite response to sulfate under butyrate amendment. Although OTU0062 responded stronger in sulfate-stimulated as compared with -unstimulated microcosms, the opposite response was observed for OTU0029 (Figure 4). OTU0029 dominated the response under butyrate without sulfate amendment, reaching at the end of the incubation an average relative genome abundance of 1.1% and an average relative ribosome abundance of 12.9%. In comparison, in butyrate incubations with sulfate amendment OTU0062 reached an average relative genome abundance of 0.4% and an average relative ribosome abundance of 5.0%.

To assess the possibility that *Telmatospirillum* OTU0062 could represent a so far unrecognized SRM, we performed growth experiments with *T. siberiense*, which is the only cultured species of this genus (97.2% sequence similarity). *T. siberiense* was previously reported to ferment various dicarboxylic acids, pyruvate and glucose under anoxic conditions. It was further tested negative for sulfate reduction, but only with acetate as electron donor (Sizova *et al.*, 2007). Here, we show that *T. siberiense* is also unable to grow anaerobically with either formate, propionate, lactate or butyrate in the presence of sulfate as electron acceptor (Supplementary Figure S9). In addition, all attempts to amplify the SRM marker genes *dsrAB* from DNA extracts of *T. siberiense* using specific PCR assays failed.

## Discussion

### Low-abundance microorganisms drive a biogeochemically relevant process

In this study, we performed a series of controlled microcosm experiments that constituted a window into naturally fluctuating biogeochemical conditions in peat soils favorable for sulfate reduction. We show that SRM were clearly involved in the degradation of the intermediate fermentation products propionate, lactate and butyrate. It is possible that at least some of these substrates were not directly used by SRM but initially degraded by secondary fermenters to acetate, formate or H_2_/CO_2_ with SRM thriving on the latter. However, lower ST under formate and acetate as compared with propionate, lactate and butyrate rather indicated a direct utilization of these C3 and C4 compounds (Figure 1).

Responsive OTUs affiliated with ST were all composed of low-abundance OTUs in the native soil, most of which stayed below or around 0.1% relative genome abundance throughout the incubation period and were permanent members of the rare biosphere in our experiments. The only major exceptions were *Desulfomonile* OTU0144 and *Telmatospirillum* OTUs 0029 and 0062, which increased in sulfate-amended incubations to maximum relative genome abundances of 0.26–0.46% (Supplementary Figures S5a and b). As all three OTUs were of low abundance in the native soil, they classify as conditionally rare biosphere members (Lynch and Neufeld, 2015). Some responsive OTUs were affiliated to the sulfate-reducing genera *Desulfovibrio*, *Desulfomonile* and *Desulfopila* (*Deltaproteobacteria*), and *Desulfosporosinus* (*Firmicutes*), and showed a strong correlation in their response to ST (*r*=0.71–0.94; Supplementary Figure S8). In addition, several responsive OTUs not related to recognized SRM were also strongly associated with ST and/or SRM (Supplementary Figure S8). Here, two OTUs were standing out, with *Telmatospirillum* OTU0062 correlating strongly under butyrate to ST (*r*=0.87) as well as to all recognized SRM (*r*=0.90–0.96) and *Rhodospirillaceae* OTU0577 correlating to ST under propionate, lactate and butyrate (*r*=0.83–0.89). In the light of the large diversity of novel *dsrAB* variants, which have been repeatedly detected in the analyzed peatland (Loy *et al.*, 2004; Pester *et al.*, 2010; Steger *et al.*, 2011), we speculated whether these OTUs may represent so far unrecognized SRM (detailed in Supplementary Results). Because *Telmatospirillum* OTU0062 had the highest 16S rRNA identity to a cultivated species, we used the type species *T. siberiense* as a case example to evaluate this hypothesis. However, *T. siberiense* tested negative for growth under sulfate-reducing conditions with all five substrates investigated in this study (Sizova *et al.*, 2007; and this study, Supplementary Figure S9) and for the presence of *dsrAB*. Hence, it seems more likely that *Telmatospirillum* OTU0062, and possibly also *Rhodospirillaceae* OTU0577, fermented the provided substrates, whereas SRM made syntrophic use of the formed formate and/or H_2_. However, this would not explain why both OTUs also responded to formate in the one microcosm that showed pronounced ST. As such, it may also be possible that these OTUs represent anaerobic sulfur oxidizers. As sulfide oxidation in wetlands can be coupled to the reduction of Fe(III) (Hansel *et al.*, 2015) or potentially also humic matter (Pester *et al.*, 2012b), *Telmatospirillum* OTU0062 and *Rhodospirillaceae* OTU0577 may represent microorganisms that are responsible for this so far missing link in the cryptic sulfur cycle, either by directly being involved in catalyzing these reactions or using elemental sulfur resulting from chemical oxidation of sulfide with Fe(III) compounds. Recently, correlation of sulfate-reducing activity and anaerobic CH_4_ oxidation was reported for three North American wetlands (Segarra *et al.*, 2015). We did not observe any evidence for the presence and activity of typical microorganisms mediating sulfate-dependent anaerobic CH_4_ oxidation in our incubations, despite the good coverage of the used primers for ANME archaea (ANME-1: 77% ANME-2a/2b: 89% ANME-2c: 76% and ANME-3: 82% Quast *et al.*, 2013) and the detection of related archaea within the orders *Methanosarcinales* (five OTUs) and *Methanocellales* (five OTUs).

### A novel mechanism to display metabolic activity

The relationship between non-growth activity and cellular rRNA content is not understood but important for the interpretation of environmental rRNA data to characterize microbial communities (Blazewicz *et al.*, 2013). Our results provide strong evidence that a rare *Desulfosporosinus* species can follow an ecological strategy to increase its cellular rRNA content while maintaining its population size over a period of 50 days. As *Desulfosporosinus* OTU0051 always correlated best in its ribosome response to ST among all responsive SRM (*r*=0.89–0.94) and contributed the highest relative ribosome abundance of this metabolic group, our results strongly suggest that this increase in protein synthesis potential was also translated into metabolic activity. All microorganisms have to divide at some time in their lives to avoid extinction. Population growth of a *Desulfosporosinus* species was observed in a DNA-stable isotope probing study of the same peatland after a longer period of incubation (73 days) (Pester *et al.*, 2010). Direct comparisons between the two studies are difficult because of the different incubation conditions; that is, peat soil microcosm incubations with a substrate mixture of formate, acetate, lactate and propionate, and two starvation phases in the previous study (Pester *et al.*, 2010). Whether the addition of a substrate mixture, especially in the presence of acetate that is a commonly supplemented building block for biomass production in anoxic cultivation media, was responsible for the observed growth remains uncertain, but may explain the different growth response as compared with this study. The previous study also showed that *Desulfosporosinus* stayed rare in snapshot samples of the native soil spanning over a time period of 3 years. The combined results of this and the previous study thus indicate that rare peatland *Desulfosporosinus* species (i) maintain a stable, low-abundance population in the studied peatland and (ii) respond to favorable environmental conditions first by increasing their ribosome content and metabolic activity and only after prolonged time by growth. This is supported by the fact that *Desulfosporosinus* species contain an exceptionally high number of ribosomal (*rrn*) operons in their genomes (8–10 copies; Pester *et al.*, 2012a), which would enable them to react very quickly to favorable conditions by an increase in ribosomes. This strategy would make sense in the highly fluctuating conditions of peatlands in space and time (Jacks and Norrström, 2004; Knorr *et al.*, 2009; Reiche *et al.*, 2009; Frei *et al.*, 2012) and would be beneficial in escaping grazing pressure or viral attack (Lynch and Neufeld, 2015). In addition, such a strategy may be widespread in different habitats as suggested by the high ribosome to genome ratios of certain rare biosphere members in a 3-year survey of marine bacterioplankton (Campbell *et al.*, 2011) and in a snapshot analysis of a hypersaline lake sediment (Weigold *et al.*, 2015).

Rare biosphere members are either considered as conditionally rare taxa that eventually grow to large population sizes upon favorable conditions or as permanently rare taxa that express rapid, periodic small abundance changes upon activity (Lynch and Neufeld, 2015). Here, we provide conclusive evidence that the *Desulfosporosinus* species in the studied peatland soil is prototypical of a new strategy of rare biosphere members, namely to postpone growth and remain at a constant low population size while maintaining increased metabolic activity over prolonged time spans. As *Desulfosporosinus* species are members of the rare biosphere in permafrost soil, natural wetlands and rice paddies worldwide (Supplementary Figure S10), their sulfate-reducing activity and ecological strategy may have a widespread effect on ecosystem functioning in these environments. In conclusion, our results underscore the importance of the rare microbial biosphere not only as a seed bank of dormant microorganisms but also as an active mediator of biogeochemical processes that buffer against climate change.
