# Supplementary material for: Consortia of low-abundance bacteria drive sulfate reduction-dependent degradation of fermentation products in peat soil microcosms
Source: ISME J. 2016 Mar 25;10(10):2365–75. doi: 10.1038/ismej.2016.42 (PMC4930147; doi:10.1038/ismej.2016.42)
Supplement: Supplementary Information [file ismej201642x1.pdf]

Consortia of low-abundance bacteria drive sulfate reduction-dependent degradation of fermentation products in peat soil microcosms

Supplementary Information

Bela Hausmann, Klaus-Holger Knorr, Katharina Schreck, Susannah G. Tringe, Tijana Glavina del Rio, Alexander Loy, Michael Pester

Supplementary Methods

Soil sampling site

The acidic peatland Schlöppnerbrunnen II is a long-term experimental field site in southeastern Germany (50°07′54.8″N, 11°52′51.8″E, 713 m above sea level) and has an average pH of 4–5 (Loy *et al.*, 2004; Küsel *et al.*, 2008; Reiche *et al.*, 2009). A detailed description of this peatland was provided previously (Matzner, 2004; Paul *et al.*, 2006). *In situ* concentrations of sulfate and short-chained fatty acids as well as lactate are generally low (on average <300 µM; Loy *et al.*, 2004; Schmalenberger *et al.*, 2007; Küsel *et al.*, 2008; Reiche *et al.*, 2009). Fresh soil was sampled for molecular analyses and sulfate/substrate turnover measurements in September 2010 and for determination of CO_2/CH_4 production in September 2011. Sampling was performed with a box-corer (10 × 10 cm; Jeglum *et al.*, 1992) and fractionated into 10 cm-depth sections. Peat water was retrieved at the same time. Samples were transported in a cooler to the laboratory and stored at 4 °C for one week before microcosms were set up. In addition, three replicate soil samples of 10–20 cm depth were frozen at −80 °C until nucleic acid extraction (native soil, i.e., the seed community). An additional soil core was sampled in April 2013 for determination of the soil water content. Peat soil was dried at 60 °C until weight loss by water evaporation could not be detected any more (n=3). Water content was 78% as determined by calculating the difference in wet and dry soil weight.

Microcosm sampling

After periodic sulfate and substrate amendments, soil slurries (microcosms) were mixed by shaking and 300 µL of the supernatant was immediately sampled and stored at −20 °C for determination of sulfate and substrate concentrations. For molecular analyses, approximately 1.5 mL of the soil slurry was sampled one day after sampling of the supernatant, flash frozen in liquid N_2, and stored at −80 °C until nucleic acids extraction. Amplicon sequencing and qPCR was performed with samples from days 5, 26, and 50. Additional samples from day 15 were analyzed by qPCR. During soil sampling, the headspace of each microcosm was replaced by 100% N_2 to reduce H_2S accumulation. The pH of all microcosms was measured before and after the incubation period. The pH at the starting point of the incubation was 4.1. After 53 days of incubation, the pH increased slightly in all microcosms ranging from 4.2 to 5.0. One of the sulfate- and butyrate-amended microcosms was lost after soil sampling at day 43. DNA and complementary DNA (cDNA) samples from this time point were analyzed together with day 50 samples from the other two replicates of this treatment. A detailed overview of the incubation and sampling strategy is given in Supplementary Figure S1.

Quantification of CO_2 and CH_4 emissions

Microcosms to follow CO_2 and CH_4 production were set up in sterile 100 mL bottles as described in the main text and incubated for 27 days only. 1.5 mL of the headspace were sampled weekly (days 0, 4, 11, 18, 25, 27) and replaced by 100% N_2. CO_2 and CH_4 were quantified with an SRI 8610C gas chromatograph (SRI Instruments, Torrance, CA, USA) equipped with a Hayesep D column, a flame ionization detector, and a carbon dioxide methanizer. Dissolved gases were calculated using Henry's law and added to the measured amount in the headspace. Rates of methanogenesis and CO_2 production were calculated from the difference in gas production between the last three time points (days 18 to 27). The share of sulfate reduction to total CO_2 production was estimated based on the ST of the last three measured time points under each separate substrate (days 21 to 27) and the assumption of complete substrate oxidation (assuming that equivalents of acetate are completely oxidized to two CO_2 with the eight released reducing equivalents being used to reduce sulfate to sulfide).

Nucleic acid extraction

Soil samples were ground in liquid N_2 and separated into approx. 300 mg aliquots, followed by total nucleic acids extraction as described previously (Leininger *et al.*, 2006). Nucleic acids were purified with the OneStep PCR inhibitor removal kit (Zymo Research, Irvine, CA, USA) and split into two fractions. Each fraction was either treated with RNase (RNase ONE ribonuclease, Promega, Fitchburg, WI, USA) followed by sodium acetate precipitation (Sambrook and Russell, 2001) or DNase (TURBO DNA-free kit, Thermo Fisher Scientific, Waltham, MA, USA) to obtain RNA-free DNA or DNA-free RNA, respectively. PicoGreen dsDNA and RiboGreen RNA assays (Thermo Fisher Scientific) were used for nucleic acids quantification. cDNA was obtained using the SuperScript III first-strand synthesis kit (Thermo Fisher Scientific) with either random hexamer primers for subsequent amplicon sequencing or the primer DSP821R (Pester *et al.*, 2010) for subsequent qPCR analysis of *Desulfosporosinus* 16S rRNA (Pester *et al.*, 2010).

16S rRNA gene and cDNA amplicon sequence data processing

34.4 million sequences were obtained from the native soil and all microcosm incubations at days 5, 26, and 50. Quality control, trimming, paired-end assembly, and clustering of obtained MiSeq reads was performed with the iTagger pipeline version 1.1 using the default 16S rRNA V4 region configuration (http://bitbucket.org/berkeleylab/jgi_itagger/). In short, DUK (http://duk.sourceforge.net/) was used for removal of PhiX control sequences, sequencing library adapter dimers, and other known contaminants (for example, human sequences), followed by trimming of the PCR primer with cutadapt (Martin, 2011). Paired-end assembly was performed using FLASH (Magoč and Salzberg, 2011) and non-merged/single-end reads were discarded. Final quality control consisted of filtering with a cutoff of 2 errors (below defined quality threshold) per 100 nt. All sequences represented by at least two identical reads were used for clustering into operational taxonomic units (OTUs) using UPARSE (Edgar, 2013). To avoid insufficient clustering, an iterative approach was applied by using a 99%, then 98%, and then 97% sequence identity threshold. Singletons that were removed in previous steps were assigned to existing OTUs by iTagger, if possible, or discarded. Reference-based chimera filtering was performed with UCHIME (Edgar *et al.*, 2011). 5.4%, 27.9%, 4.5%, 0.1% of all reads were removed during quality control, UPARSE clustering (including *de novo* chimera filtering), singletons filtering, and reference-based chimera filtering using UCHIME, respectively. Alpha diversity metrics (observed OTUs, Chao1, ACE, and Good's coverage) were determined (Supplementary Figure S3a) using R (R Core Team, 2015) and phyloseq (McMurdie and Holmes, 2013).

Relative genome abundance for each OTU and sample was estimated by correcting 16S rRNA gene abundance for *rrn* operon copy number bias. *rrn*DB 4.3.3 (Stoddard *et al.*, 2015) was extracted in R and the mean *rrn* operon copy number for *Desulfosporosinus* was updated to 8.75 based on new information (Pester *et al.*, 2012a). *rrn*DB provides mean *rrn* operon copy numbers for taxonomic ranks ranging from domain to genus, which were assigned to each OTU based on the best matching RDP classification rank. 22 OTUs that could not be classified at domain level by the RDP classifier were assigned an arbitrary *rrn* operon copy number of 1. The other extreme were two OTUs assigned to *Brevibacillus* with an *rrn* operon copy number of 15. Most OTUs were assigned *rrn* operon copy number between 1.37 and 4.43 (10% and 90% quantiles, respectively). The *rrn* operon copy numbers assigned to the responsive OTUs were 3.83 (OTU0029), 8.75 (OTU0051), 3.83 (OTU0062), 1.00 (OTU0144), 3.57 (OTU0167), 3.57 (OTU0200), 8.75 (OTU0228), 3.50 (OTU0256), 2.12 (OTU0258), 3.83 (OTU0273), 2.00 (OTU0339), 5.50 (OTU0346), 2.12 (OTU0467), 6.53 (OTU0487), 3.83 (OTU0577), 2.12 (OTU0793), 3.00 (OTU0924), 2.12 (OTU0950), 2.12 (OTU0955), 3.77 (OTU0999), 3.10 (OTU1007), 2.12 (OTU1066), 3.83 (OTU1078), 4.43 (OTU1151), 2.96 (OTU1244), 2.12 (OTU1303), 1.00 (OTU1405), 3.83 (OTU1641), 4.00 (OTU1710), 2.12 (OTU1809), 2.00 (OTU1963), 2.96 (OTU1998), 1.37 (OTU2005), 2.12 (OTU2028), and 5.50 (OTU2131).

Phylogenetic tree reconstruction

The phylogeny of selected OTUs was analyzed by calculating trees in ARB (Ludwig *et al.*, 2004). Reference sequence RAxML trees were inferred based on 817 (*Telmatospirillum*) or 843 (*Desulfosporosinus*) alignment positions without applying a conservation filter (Stamatakis, 2014). Tree topology was evaluated by bootstrap analysis (100 resamplings) with RAxML (Stamatakis, 2014). Shorter V4 amplicon sequences were added to the reference trees by using ARB's parsimony interactive tool.

Statistical analysis

Data analysis was performed using R 3.2, and the R packages data.table 1.9 (https://github.com/Rdatatable/data.table/), magrittr 1.5 (https://github.com/smbache/magrittr), plyr 1.8 (Wickham, 2011), and phyloseq 1.12 (McMurdie and Holmes, 2013). Plots were generated in R using the packages ggplot2 1.0 (Wickham, 2009) and igraph 0.7 (Csárdi and Nepusz, 2006). The package edgeR 3.10 (Robinson *et al.*, 2010) was used to test for differential abundance patterns (benchmarked for 16S rRNA gene OTU data by McMurdie and Holmes, 2014). Independent pairwise comparisons were performed between different treatments and time points using edgeR's general linear model approach. Per OTU, three different types of differential abundance tests were performed. (1) Sulfate effect: for each substrate and time point, sulfate-amended microcosms were compared to their corresponding non-stimulated controls (i.e., did the tested OTU respond to external sulfate stimulation). (2) Substrate effect: substrate-amended microcosms were compared to the no-substrate-controls. This was done separately for each time point and substrate under either sulfate-stimulated or unstimulated conditions (i.e., under which substrate-amendments did the tested OTU respond). (3) Temporal effect: within each treatment, the 26 d and 50 d time points were compared to the 5 d time point (i.e., did the relative 16S rRNA (gene) abundance of the tested OTU increase in time). Pairwise comparisons consisted of three replicates each, with the exception of the propionate- and sulfate-amended microcosms, where one replicate was excluded from all analyses because of its inconsistent sulfate turnover (Figure 1). Before each differential abundance test, OTUs were filtered. To be included, OTUs had to be reliably detected, i.e., be represented by more than 10 reads in at least two out of six samples. This resulted in the statistical testing of 1491 out of 7435 OTUs.

OTUs were considered responsive if all three types of differential abundance tests were significant (FDR-corrected p-value <0.05). OTUs significantly more abundant under sulfate-stimulated than under unstimulated conditions were denoted as sulfate-stimulated responders. Inversely, OTUs significantly more abundant in the no-sulfate microcosms were denoted as sulfate-deterred responders. Each response was always assigned to a certain substrate (significant substrate effect) and could be an intermediate response (after 25 days), and late response (after 50 days), or both (significant temporal effect). We also tested for sulfate-stimulation and time-dependent response with endogenous substrates by omitting the substrate effect test in the no-substrate-control microcosms.

For snapshot analyses of environmental 16S rRNA using only one time point, it has been pointed out before that the amount of ribosomal RNA is not only influenced by current conditions but also by life history (past) or ecological strategy (future) (Blazewicz *et al.*, 2013). Our experimental design largely excluded such effects. Since we tested for increases in ribosome abundance over time, a legacy from past activity expressed as a constantly high ribosome content (for example, Sobek *et al.*, 1966) could be excluded. An increase of the ribosome content by inactive populations entering starvation and dormancy (for example, Sukenik *et al.*, 2012) could be excluded as well by omitting OTUs that responded unspecifically, i.e., irrespective of sulfate and/or specific substrate additions.

qPCR targeting the genus *Desulfosporosinus* and *Bacteria/Archaea*

qPCR assays targeting the 16S rRNA genes of the genus *Desulfosporosinus* and most *Bacteria* and *Archaea* were performed as described previously (Pester *et al.*, 2010). Primer 1389F was modified to cover more *Archaea* (5′-TGY ACA CAC CGC CCG T-3′). In addition we analyzed the 16S rRNA cDNA of the genus *Desulfosporosinus* by reverse transcription using the primer DSP821R, followed by qPCR (Pester *et al.*, 2010). To estimate *Desulfosporosinus* ribosome and genome abundance per cm^3 of fresh soil, 16S rRNA (gene) copies per ng of extracted DNA/RNA were normalized against the amount of soil used for nucleic acid extraction, based on following assumptions: 100% nucleic acids extraction efficiency, 100% reverse transcription efficiency, *Desulfosporosinus rrn* operon copy number of 8.75 (Pester *et al.*, 2012a), a soil bulk density of 0.29 g cm^{-3} (Goldberg *et al.*, 2008), and a water content of 78% (this study). Total *Bacteria* and *Archaea* gene copy numbers per cm^3 of fresh soil were calculated analogous.

qPCR of species-level *dsrA* OTUs

Abundances of the two *dsr*AB OTUs 1 and 2 in the incubations were measured by using established *dsr*A-targeted qPCR assays (Steger *et al.*, 2011). Gene copy numbers per cm^3 of fresh soil were calculated analogous to the qPCR assay for *Desulfosporosinus*.

Global distribution and abundance of *Desulfosporosinus* in wetlands

We gained insights into the global distribution and abundance of *Desulfosporosinus* species by identifying sequences in public databases with ≥97% 16S rRNA identity to the *Desulfosporosinus* OTUs 0051 and 0228. We used blastn (Camacho *et al.*, 2009) and the 253 nt long OTUs sequences to query GenBank (Benson *et al.*, 2013). To identify closely related sequences in the short read archive (SRA) (Leinonen *et al.*, 2011), we initially choose proxy sequences for each OTU that were >1400 nt long and 100% identical over the V4 region of the 16S rRNA (GenBank accession numbers EU981233 and KJ650684, respectively). This made it possible to survey sequence information of non-V4 region amplicon data sets. We applied these proxy sequences in the integrated microbial NGS platform (http://www.imngs.org/) to retrieve all SRA samples that harbored ≥97% identical reads. The metadata of all GenBank and SRA matches was manually screened to identify all sequences that originated from wetlands (including rice paddy and permafrost soils). If sufficient metadata was available, we classified source environments into peatland, permafrost, rice paddy, or other soils. We distinguished between sequences obtained directly from soils (i.e., pristine soils, rice paddy fields, or restored wetlands) or from soils that were manipulated (for example, microcosms incubations, SIP enriched samples, or enrichments/isolates). Locations of samples without available geographical coordinates were estimated based on other information given (for example, country/region of origin). For all demultiplexed samples, we also retrieved the relative abundance of the 16S rRNA gene reads that were closely related (≥97% sequence identity) to the two *Desulfosporosinus* OTUs.

Supplementary Results

CO_2 and CH_4 production

We used a second set of microcosms that were identical in incubation conditions to the setup used for the molecular analyses to monitor total CO_2 and CH_4 production under the various substrate and sulfate scenarios. The amount of total organic carbon mineralized (sum of produced CO_2 and CH_4) in the individual microcosms as well as the fraction of organic carbon added by external substrates is presented in Supplementary Table S1. Surprisingly, added substrates did not result in a surplus amount of organic carbon mineralization. A possible explanation could be that microorganisms using either internal or external substrates competed for the same limiting factor, which was however not the electron donor. All provided substrates are most likely easier to degrade than internal substrates, which have to be extracted from humic matter. Short-chained fatty acids and lactate can be only degraded by anaerobic respiration (for example, using nitrate, Fe(III), or sulfate as electron acceptor) or by secondary fermenters, which have to cooperate typically with methanogens and have a lower energy yield as compared to anaerobically respiring microorganisms (Schink and Stams, 2006). Therefore, a plausible limiting factor could be the various electron acceptors, especially Fe(III). It has been shown that iron reduction is an important anaerobic carbon mineralization process in the studied peatland (Küsel *et al.*, 2008). In addition, iron reduction is a primarily surface-bound process posing a potentially limiting factor to the accessibility of this electron acceptor.

At the end of the incubation time (days 18–27), CH_4 production was reduced in sulfate-supplemented microcosms by 83±9.4%, 98±1.0%, 100±2.0%, 97±3.6%, and 99±0.1% (mean and 95% confidence intervals) in the presence of formate, acetate, propionate, lactate, or butyrate, respectively. Contrary, sulfate reduction was responsible for 50±11%, 61±11%, 43±20%, 53±22%, and 102±8% of total CO_2 production at the very end of these incubations, respectively (same order of substrates as above).

In control incubations without sulfate amendment, methanogenesis accounted for 0.1–0.2% of the total carbon flow during organic matter mineralization over the total incubation time of 27 days (Supplementary Table S1), which is again most likely explained by the presence of active Fe(III)-reducing microorganisms (Küsel *et al.*, 2008). In line with these low activities, methanogenic archaea did not respond significantly by increasing their 16S rRNA or 16S rRNA genes over time in these methanogenic controls. A primer bias seems unlikely because the used primers cover 87% of all *Euryarchaeota* sequences in the ARB-SILVA database (*Methanobacteriales* 93%, *Methanococcales* 84%, *Methanocellales* 89%, *Methanomicrobiales* 91%, *Methanosarcinales* 89%, *Methanopyrales* 0% – contains only five sequences, *Thermoplasmatales* 85%; Quast *et al.*, 2013) and we detected five OTUs within the *Methanosarcinales* and five OTUs within the *Methanocellales*.

Mock community

A mock community consisting of the activated sludge clones H42, H29, H28, H13, and H44 (GenBank accession numbers AF234715, AF234692, AF234749, AF234737, and AF234743, respectively) was mixed at a ratio of 76%, 18%, 5%, 0.7%, and 0.09% relative abundance, respectively (Herbold *et al.*, 2015), and included in one of the Illumina MiSeq sequencing runs. Clones were mapped to the representative OTU sequences using blastn and applying a threshold of 97% sequence identity to accommodate potential sequencing errors, resulting in an unambiguous assignment. Log-transformed expected and measured abundances for each mock community clone were compared by fitting a linear regression (R Core Team, 2015), which resulted in a significant slope of 1.0 (p-value=0.01, R^2=0.90, Supplementary Figure S4a) and suggests that relative abundance shifts can be reliably measured by our amplicon sequencing approach. The five most abundant OTUs were assigned to the original mock community clones (Supplementary Figure S4b). The sixth most abundant OTU with only 7 reads was assigned to

Escherichia/Shigella and was most likely a contamination (Salter *et al.*, 2014). For comparison, the least abundant mock community clone had 119 reads.

Phylogenetic analysis of *Desulfosporosinus* and *Telmatospirillum* OTUs

Phylogenetic analysis of the two responsive *Desulfosporosinus* OTUs and the three responsive *Telmatospirillum* OTUs verified their placement within the genera *Desulfosporosinus* (Supplementary Figure S7a) and *Telmatospirillum* (Supplementary Figure S7b), respectively. The pairwise similarity of the two *Desulfosporosinus* OTUs was 96.8% and thus within the genus-level threshold of 94.5% (Yarza *et al.*, 2014). Interspecies similarity of *Desulfosporosinus* pure cultures reaches a minimum of 92.5%. OTU0051 is identical (over the V4 region) with eight clones from the heavy DNA fraction of sulfate-amended SIP incubations of the same peatland (Pester *et al.*, 2010). In contrast, OTU0228 had a maximum sequence similarity of 98% to clones from the SIP study.

Pairwise sequence similarities between the three *Telmatospirillum* OTUs varied between 94.5% and 96.8%. OTU0062, which responded stronger in sulfate-stimulated incubations as compared to unstimulated control incubations, was phylogenetically clearly separated from *Telmatospirillum* OTU0029, which showed the opposite response. In addition, OTU0062 was identical (over the V4 region) to 7 SIP clone sequences identified previously in the heavy DNA fraction of sulfate-amended as well as unamended SIP incubations of the same peatland (Pester *et al.*, 2010). OTUs 0029 and 0273 had a maximum sequence similarity of 95% and 96% to clones from the SIP study, respectively.

qPCR analysis of abundant species-level *dsrAB* OTUs

Previous studies of this peatland identified a large diversity of reductive-type *dsr*AB sequence variants (coding for subunit A and B of the dissimilatory sulfite reductase), which serve as functional marker genes for SRM but are also present in some sulfite reducers, degraders of organosulfonates, and syntrophic microorganisms that likely lost the capability to reduce sulfate (Müller *et al.*, 2015). In the studied peatland, so far 53 different *dsr*AB OTUs at the approximate species level were identified with most of them affiliating to deep-branching lineages with no cultured representative (Pester *et al.*, 2012b). Several of these unidentified *dsr*AB OTUs represent autochthonous and abundant members of the microbiota in this peatland (Steger *et al.*, 2011). We monitored the abundance of two of these OTUs (designated *dsr*AB OTU 1 and 2; Steger *et al.*, 2011) in our incubations by qPCR. However, there was no clear response to sulfate amendment under any of the tested substrate scenarios (Supplementary Figure S6c). These results are in line with findings from a previous DNA-SIP study, which could not detect selective ^{13}C-labeling of these novel *dsr*AB variants in sulfate-treated microcosms (Pester *et al.*, 2010). These novel deep-branching *dsr*AB lineages either derive from non-SRM or the tested substrates might not have been in the metabolic range of SRM harboring these novel *dsr*AB variants.

Supplementary Tables

Supplementary Table S1

Amount of mineralized (CO_2 + CH_4) and supplemented carbon after 27 days as observed in parallel incubations (complete time series data shown in Supplementary Figure S2c). Mean and 95% confidence intervals are given in µmol. Percentages are relative to total mineralized carbon.

Treatment	Total mineralized carbon	CO_2 produced	CH_4 produced	Total substrate (C atoms) added	Contribution of added substrate to total carbon mineralization
Control – Sulfate	393±58	392±58 (99.91%)	0.36±0.219 (0.09%)	0.0 (0.0)	0%
Formate – Sulfate	303±22	303±22 (99.86%)	0.42±0.281 (0.14%)	93.6 (93.6)	31%
Acetate – Sulfate	351±59	350±58 (99.79%)	0.75±0.533 (0.21%)	57.6 (115.2)	33%
Propionate – Sulfate	260±33	260±33 (99.94%)	0.16±0.073 (0.06%)	57.6 (172.8)	66%
Lactate – Sulfate	304±74	303±74 (99.83%)	0.51±0.270 (0.17%)	57.6 (172.8)	57%
Butyrate – Sulfate	271±12	270±11 (99.82%)	0.49±0.075 (0.18%)	57.6 (230.4)	85%
Control + Sulfate	306±48	306±48 (99.99%)	0.03±0.000 (0.01%)	0.0 (0.0)	0%
Formate + Sulfate	317±76	317±76 (99.98%)	0.08±0.018 (0.02%)	93.6 (93.6)	30%
Acetate + Sulfate	313±27	313±27 (99.99%)	0.03±0.007 (0.01%)	57.6 (115.2)	37%
Propionate + Sulfate	277±60	277±60 (99.99%)	0.02±0.001 (0.01%)	57.6 (172.8)	62%
Lactate + Sulfate	309±49	309±49 (99.99%)	0.02±0.002 (0.01%)	57.6 (172.8)	56%
Butyrate + Sulfate	273±32	273±32 (99.99%)	0.03±0.002 (0.01%)	57.6 (230.4)	84%

Supplementary Figures

Supplementary Figure S1

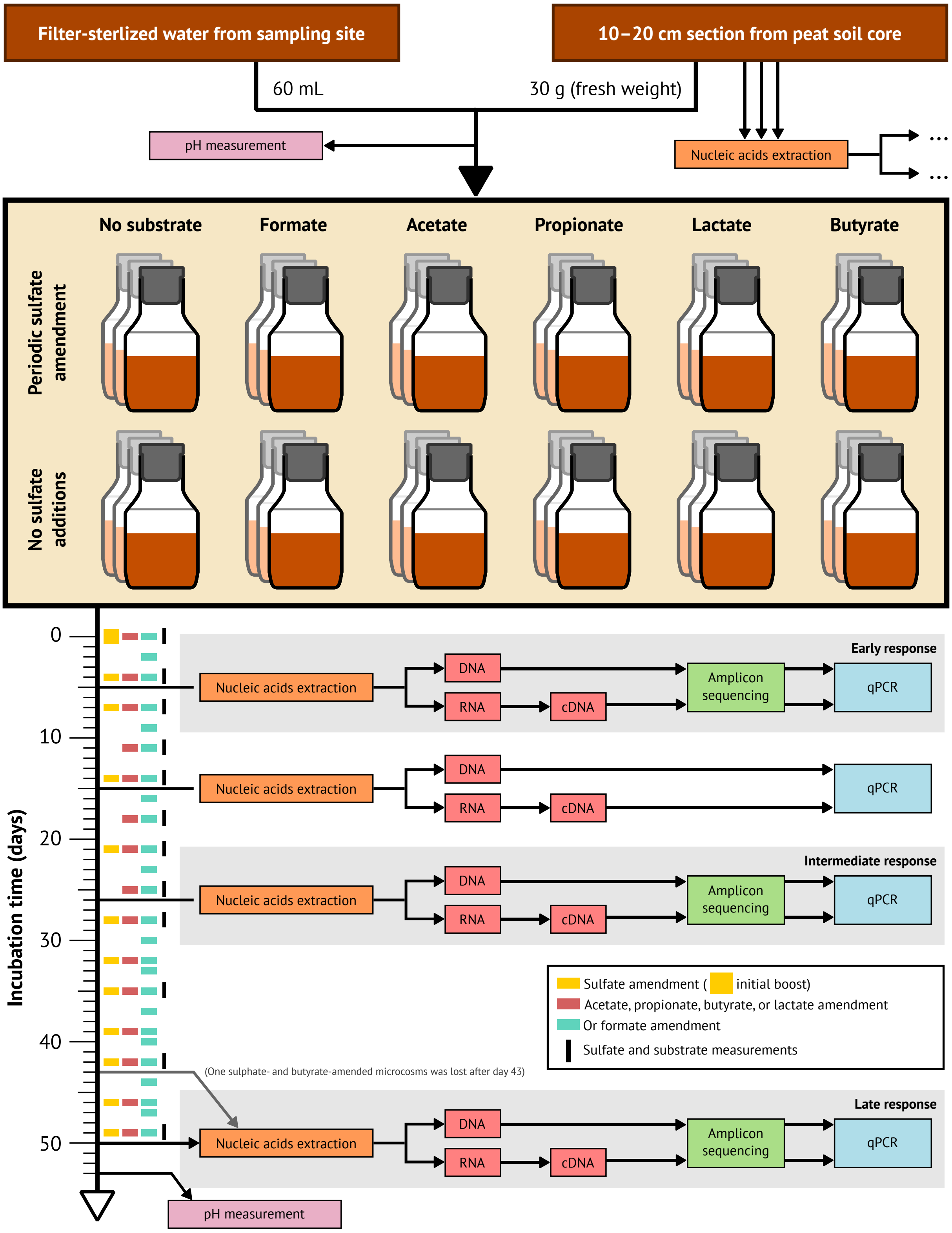

Overview of the experimental setup. Sulfate and substrate amendment regime is indicated with colored bars. Supernatant sampling for quantification of sulfate and individual substrates is indicated with black bars. Arrows indicate microcosm sampling and molecular analysis workflows or pH measurements. Additional parallel incubations for measurements of CO_2 and CH_4 are not shown. These were set up with the same sulfate and substrate amendment regime but were stopped after 27 days and gas from the headspace was sampled at days 0, 4, 11, 18, 25, and 27 to follow CO_2 and CH_4 production.

Supplementary Figure S2

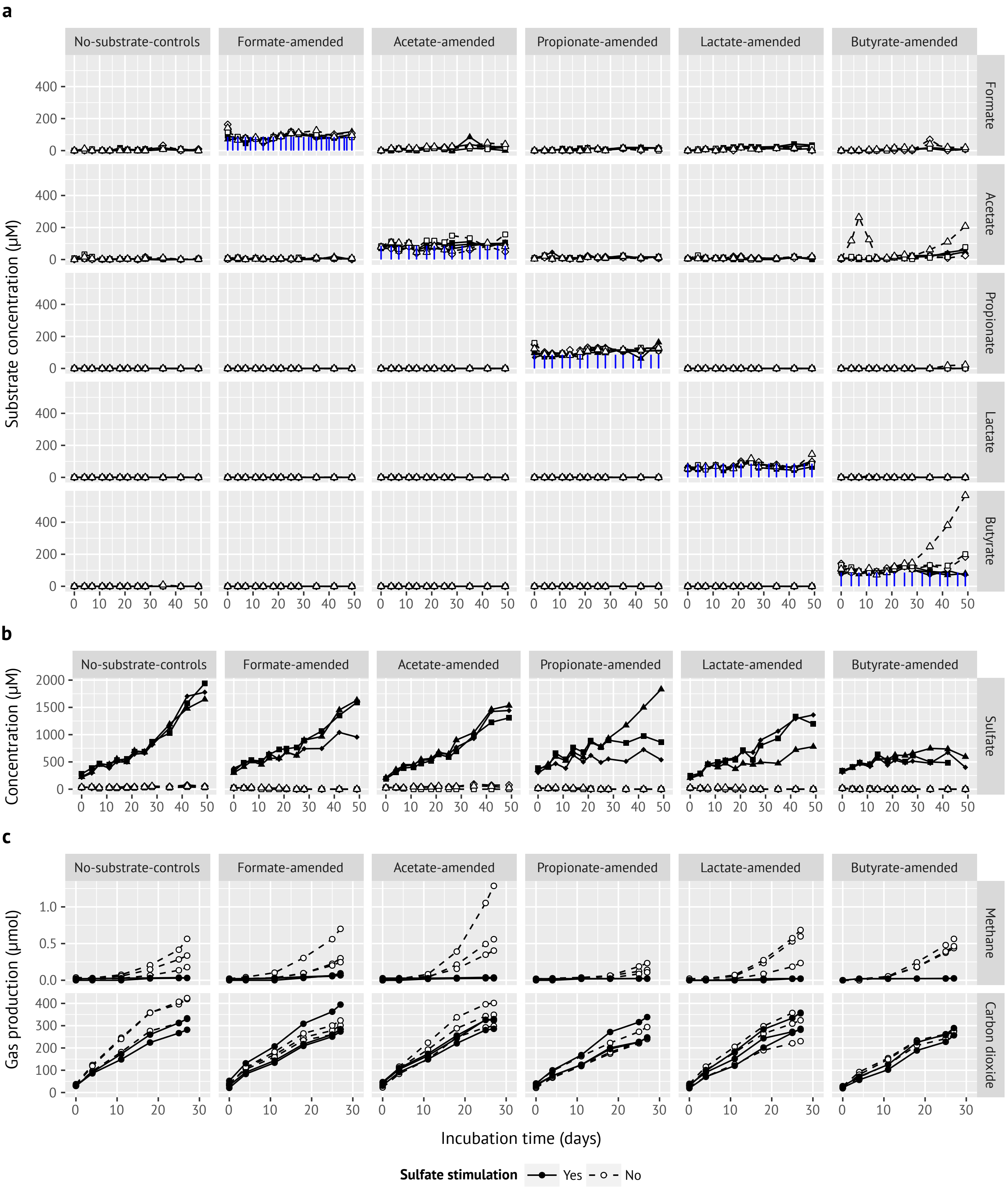

Biogeochemical characterization of peat soil microcosms. Substrate (**a**) and sulfate (**b**) concentrations (rows) as measured with capillary electrophoresis, separated by substrate treatment (columns). Solid lines and symbols depict sulfate-stimulated microcosms whereas dashed lines and open symbols depict controls without additional sulfate. Within each treatment, the shape of the symbols represents triplicate microcosms and is consistent in all figures that show data from individual replicates. Blue vertical lines represent time points where sulfate and/or substrate was amended (see also Supplementary Figure S1). Amendments were performed immediately before sampling, therefore observed concentrations should be seen as cumulative. Height of blue lines indicates approximate amounts of added substrates. (**c**) CH_4 and CO_2 production as observed in parallel microcosm incubations.

Supplementary Figure S3

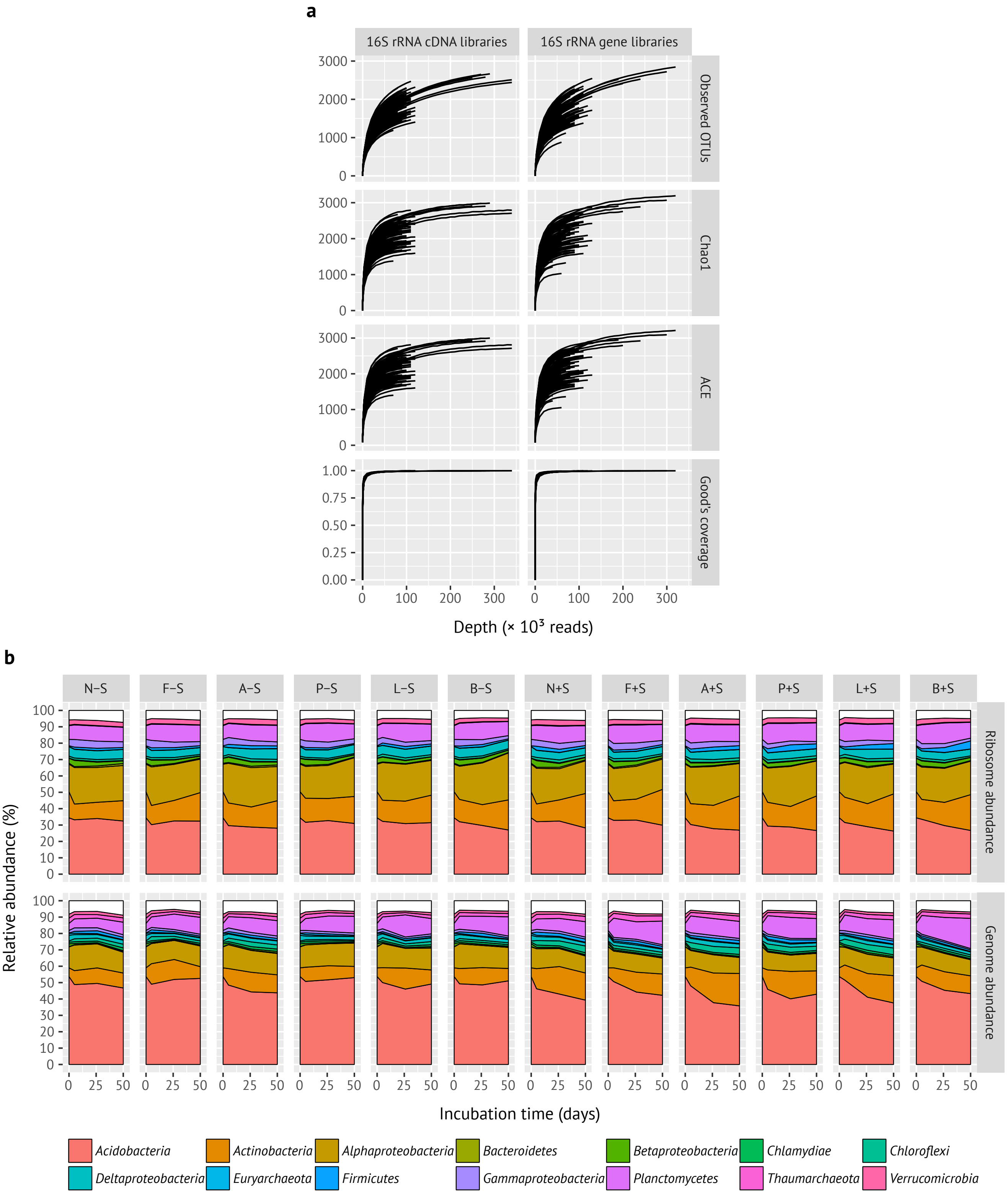

Overview of amplicon sequencing data. (**a**) Rarefaction analysis of observed OTUs, Chao1, ACE, and Good's coverage in all samples. (**b**) Temporal changes in relative ribosome and genome abundance of abundant phyla/classes in incubations with different substrates, i.e. no-substrate-controls (N), formate (F), acetate (A), propionate (P), lactate (L), and butyrate (B), respectively, and with (+S) and without (−S) sulfate stimulation. Mean relative abundances per treatment and time point are displayed as stacked area plots. Only phyla/classes with a mean abundance of over 1% in any treatment are displayed in color. Data points at day 0 depict the abundance in the native soil, which was sampled from parallel peat soil subsamples.

Supplementary Figure S4

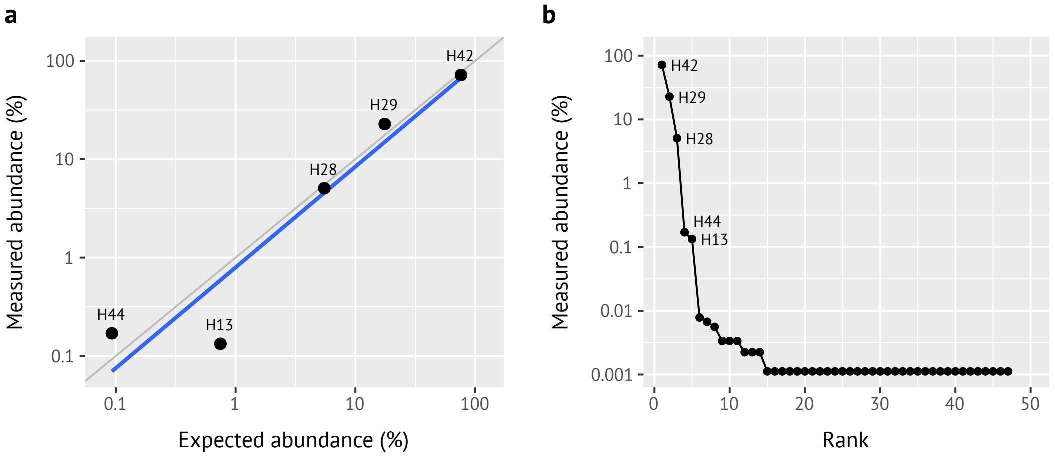

Analysis of the mock community used as a control for amplicon sequencing. (**a**) Comparisons of expected and measured 16S rRNA gene abundances of all five mock community clones. Blue line: fitted linear regression (slope=1.02, y-intercept=−0.05). Gray line: perfect fit (slope=1, y-intercept=0). Both axes are log scaled. (**b**) Rank abundance curve of the mock community. All detected OTUs are shown as points. OTUs assigned to mock community clones are indicated by their names.

Supplementary Figure S5

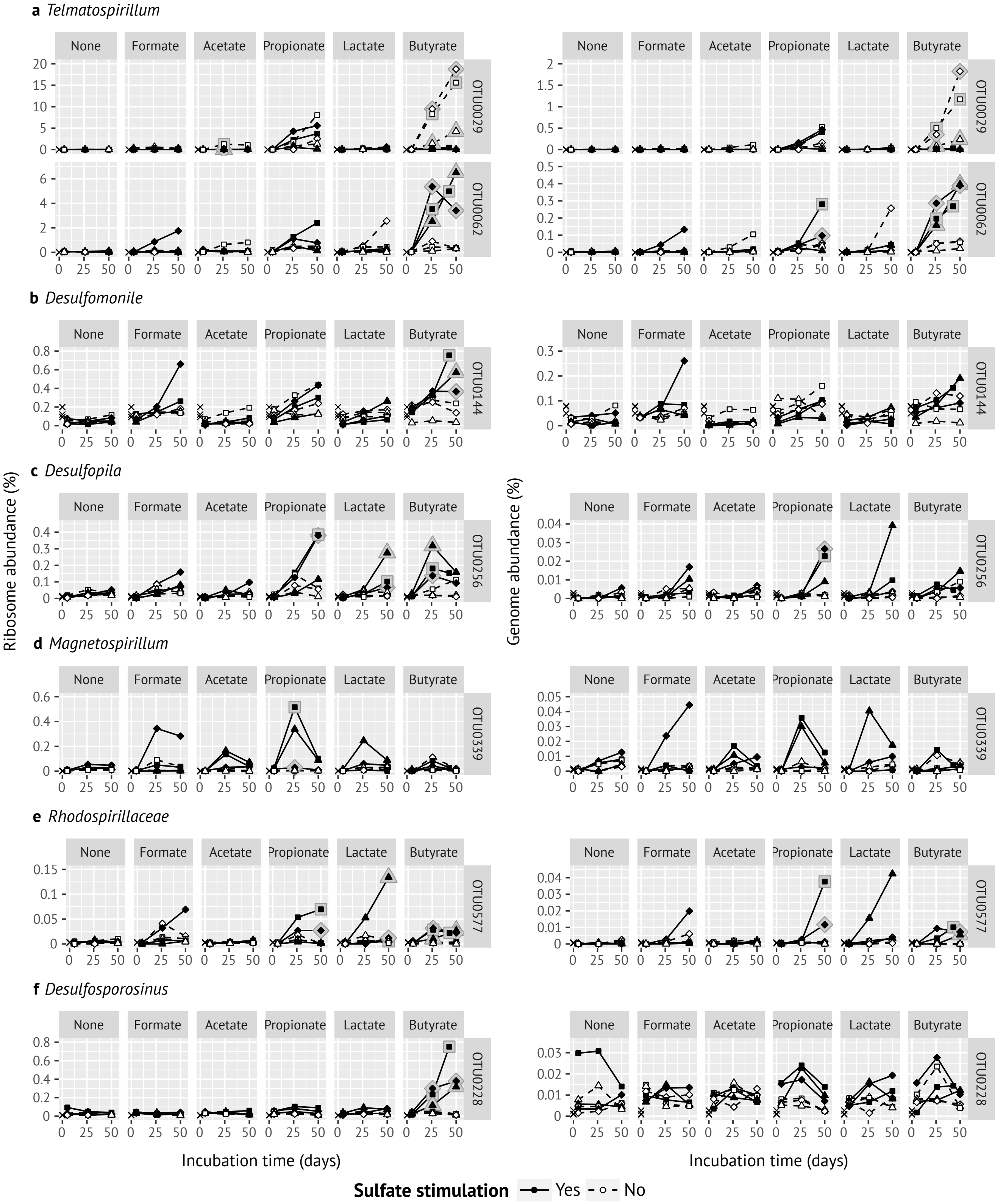

Temporal changes in relative ribosome and genome abundance of selected responsive OTUs (i.e. mentioned in the main text) in incubations with different substrates. OTUs are sorted by their number. Solid lines and symbols depict sulfate-stimulated microcosms whereas dashed lines and open symbols depict controls without additional sulfate. Diagonal crosses depict the abundance in the native soil, which was sampled from parallel peat soil subsamples and plotted at day 0 in all panels. Halos around symbols indicate significantly higher abundance in the unstimulated or sulfate-stimulated microcosms, respectively, as compared to their respective controls and day 5. Data points drawn directly on the x axis represent relative abundance values of zero.

Supplementary Figure S6

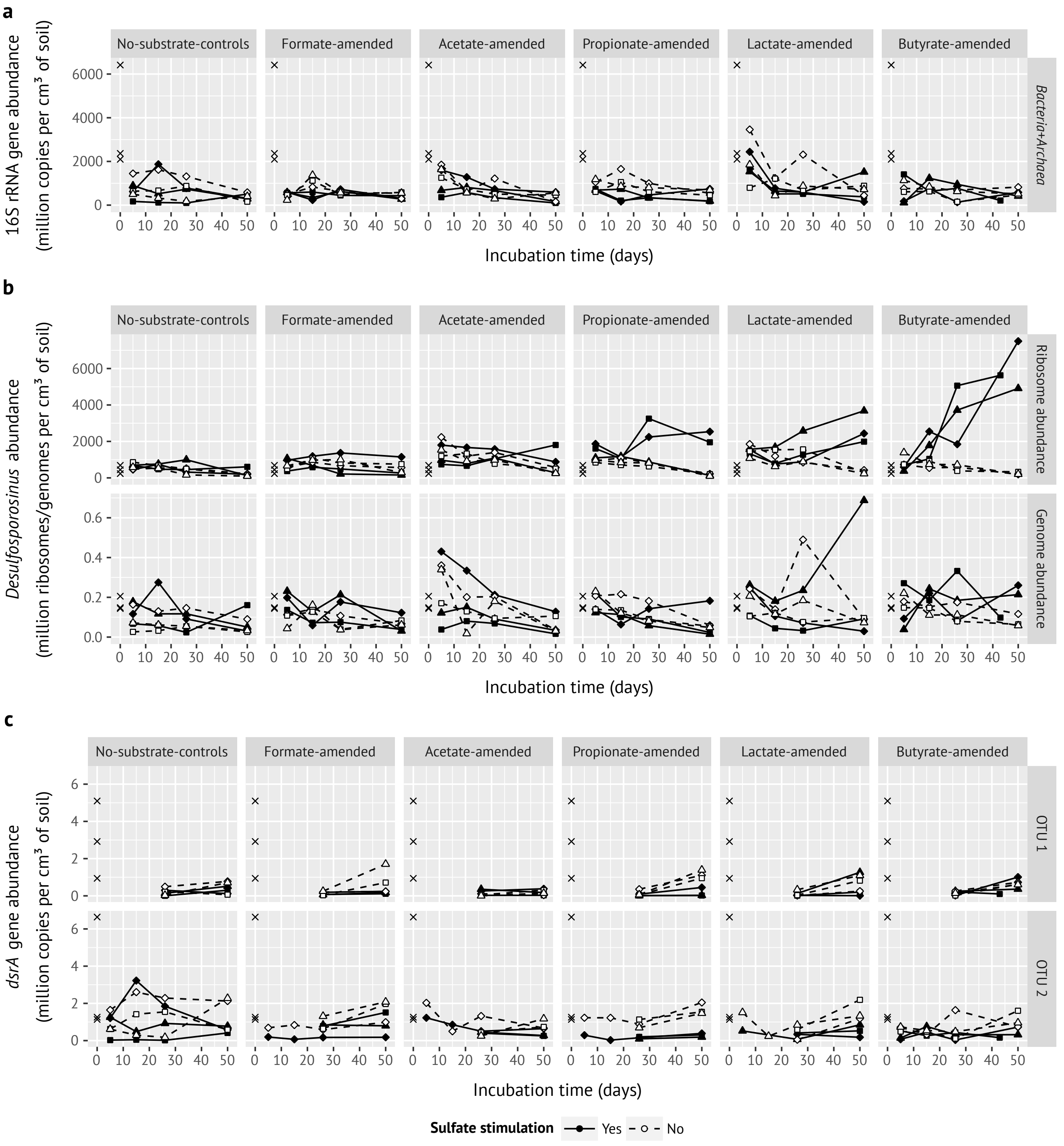

Absolute abundances of most *Bacteria* and *Archaea* (**a**), the genus *Desulfosporosinus* (**b**), and *dsr*A-OTUs 1 and 2 (**c**) in incubations with different substrates, as revealed by qPCR. Solid lines and symbols depict sulfate-stimulated microcosms whereas dashed lines and open symbols depict controls without additional sulfate. Diagonal crosses depict the abundance in the native soil, which was sampled from parallel peat soil subsamples and plotted at day 0 in all panels.

Supplementary Figure S7

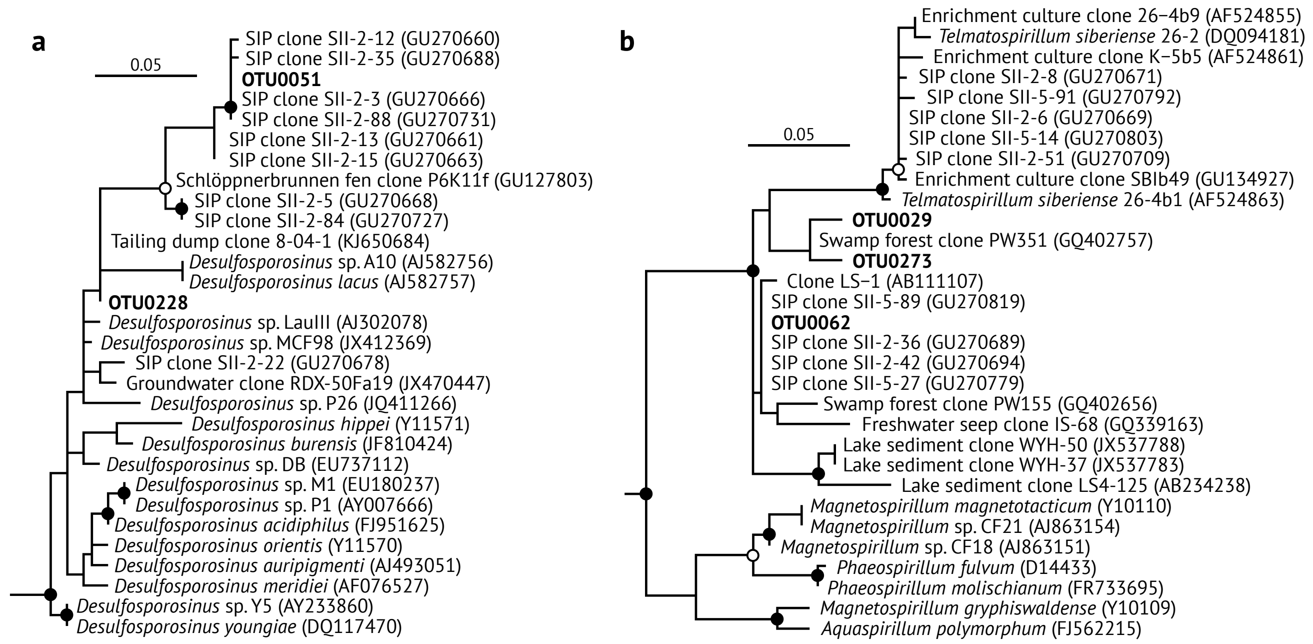

Maximum likelihood 16S rRNA trees of the genera *Desulfosporosinus* (**a**) and *Telmatospirillum* (**b**). Representative V4 amplicon sequences (bold) of identified *Desulfosporosinus* and *Telmatospirillum* OTUs were added to the tree by using ARB's Parsimony Interactive tool. Solid circles indicate ≥90% and open circles indicate ≥70% bootstrap support. The bars represent 0.05 inferred sequence divergence. GenBank accession numbers of reference sequences are given in parentheses. All SIP clones (Pester *et al.*, 2010), clone SBIb49 (Lüdecke *et al.*, 2010), and clone P6K11f (unpublished) are from the same peatland as the OTUs identified in this study.

Supplementary Figure S8

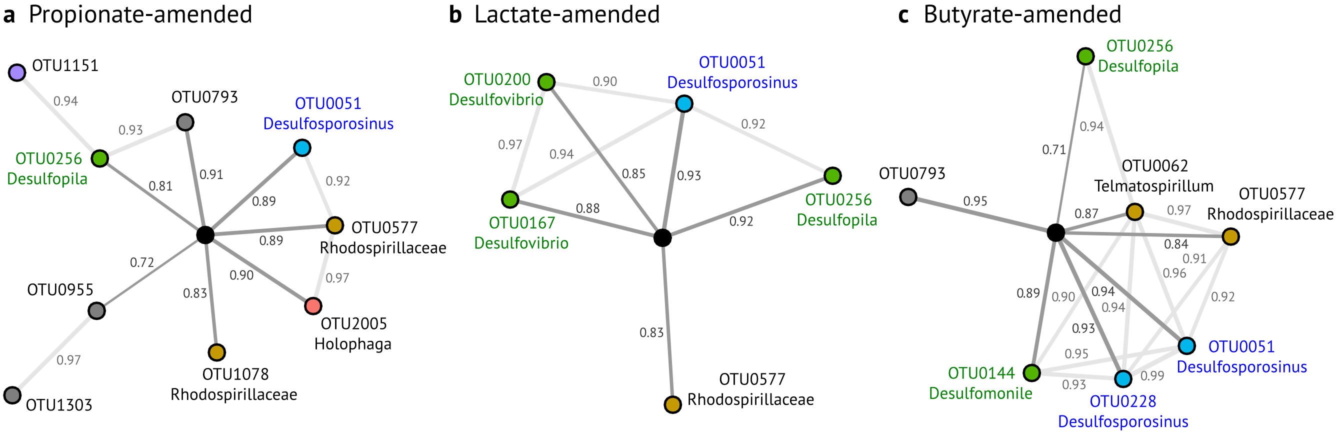

Substrate-specific interaction networks of sulfate-stimulated OTUs. Association networks visualizing correlation strength between 16S rRNA-based responses of sulfate-stimulated OTUs (colored circles, see Figure 2, SRM highlighted) among each other ($r \geq 0.9$, light gray edges) and to sulfate turnover rates (black circle, $r \geq 0.5$, dark gray edges) in (**a**) propionate-, (**b**) lactate-, and (**c**) butyrate-amended incubations with sulfate. Correlation coefficients are written next to edges.

Supplementary Figure S9

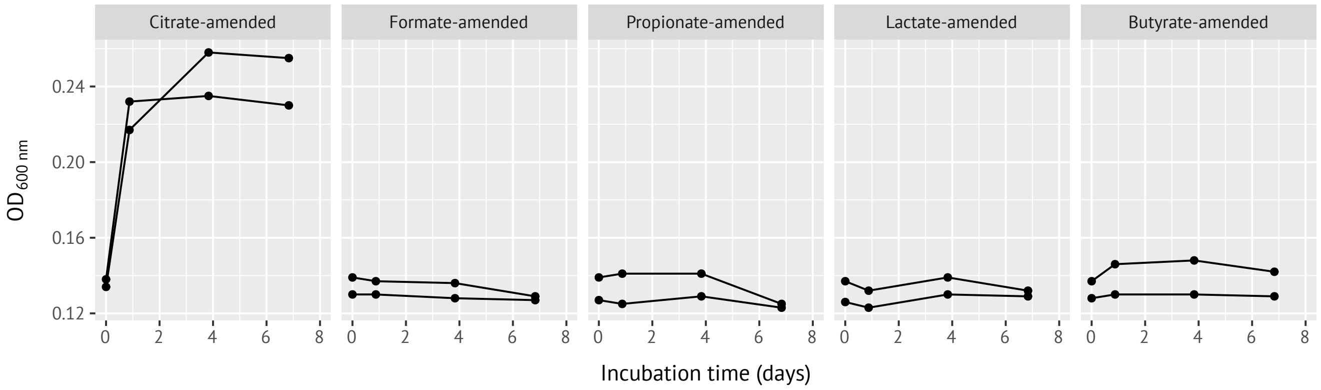

Anaerobic growth of *Telmatospirillum siberiense* 26-4b1[T] at various substrates (each 5 mM) in the presence of 1 mM sulfate. Optical densities at 600 nm are provided for two parallel incubations per substrate.

Supplementary Figure S10

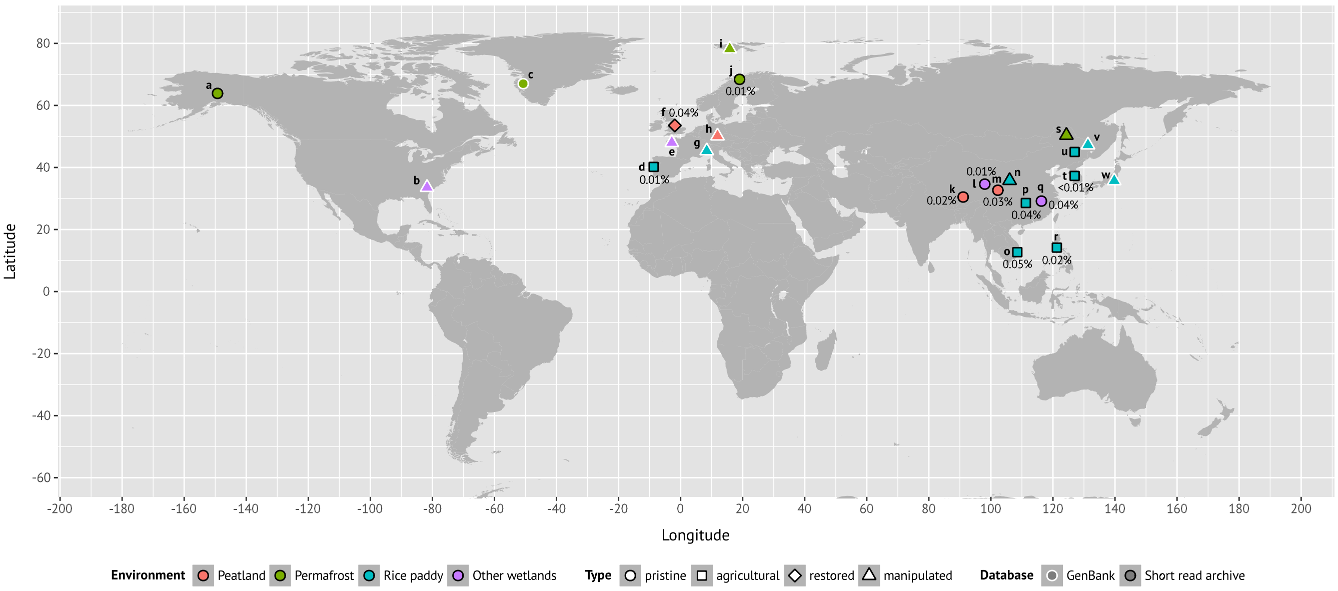

Global distribution of *Desulfosporosinus* OTUs 0051 and 0228 and their close relatives. Geography of 97% sequence identity matches in NCBI's GenBank (white-framed symbols) and the SRA (black-framed symbols) repositories. Matches are further separated by source environment (color) and study type (shape). Percentages indicate relative 16S rRNA gene abundance, if available. The maximum abundance is shown when multiple abundance values were available from multiple samples of the same site or a match of both OTUs. *a–w* indicates SRA study or GenBank accession numbers including relevant publications. *a* SRP034636 (Deng *et al.*, 2015). *b* NR_115694 (*Desulfosporosinus youngiae*; Lee *et al.*, 2009). *c* HQ625530. *d* SRP018527. *e* HE804547, HE804573, and HE804583. *f* SRP048856 (Elliott *et al.*, 2015). *g* AJ621940, AJ621942, and AJ621943. *h* same location as this study, GU127803, HG324332, HG324336, HG324560, HG325117, LK025433, and 19 clones from Pester *et al.* (2010). *i* 25 clones from Hansen *et al.* (2007). *j* SRP030775. *k–m* SRP033622 (Deng *et al.*, 2014). *n* DRP001333. *o* SRP042336 and SRP045589. *p* SRP043656. *q* SRP035329. *r* SRP047272. *s* SRP015314 (Yang *et al.*, 2012). *t* SRP013887. *u* ERP009705. *v* JX505346. *w* AB487363 (Ishii *et al.*, 2009).

Supplementary References

Benson DA, Cavanaugh M, Clark K, Karsch-Mizrachi I, Lipman DJ, Ostell J *et al.* (2013). GenBank. *Nucleic Acids Res* **41**: D36–D42.

Blazewicz SJ, Barnard RL, Daly RA, Firestone MK. (2013). Evaluating rRNA as an indicator of microbial activity in environmental communities: limitations and uses. *ISME J* **7**: 2061–2068.

Camacho C, Coulouris G, Avagyan V, Ma N, Papadopoulos J, Bealer K *et al.* (2009). BLAST+: architecture and applications. *BMC Bioinformatics* **10**: 421.

Csárdi G, Nepusz T. (2006). The igraph software package for complex network research. *InterJournal* **CX**: 1695.

Deng J, Gu Y, Zhang J, Xue K, Qin Y, Yuan M *et al.* (2015). Shifts of tundra bacterial and archaeal communities along a permafrost thaw gradient in Alaska. *Mol Ecol* **24**: 222–234.

Deng Y, Cui X, Hernańdez M, Dumont MG. (2014). Microbial diversity in hummock and hollow soils of three wetlands on the Qinghai-Tibetan plateau revealed by 16S rRNA pyrosequencing. *PLoS One* **9**: e103115.

Edgar RC. (2013). UPARSE: highly accurate OTU sequences from microbial amplicon reads. *Nat Methods* **10**: 996–998.

Edgar RC, Haas BJ, Clemente JC, Quince C, Knight R. (2011). UCHIME improves sensitivity and speed of chimera detection. *Bioinformatics* **27**: 2194–2200.

Elliott DR, Caporn SJM, Nwaishi F, Nilsson RH, Sen R. (2015). Bacterial and fungal communities in a degraded ombrotrophic peatland undergoing natural and managed re-vegetation. *PLoS One* **10**: e0124726.

Goldberg SD, Knorr K-H, Gebauer G. (2008). N_2O concentration and isotope signature along profiles provide deeper insight into the fate of N_2O in soils. *Isotopes Environ Health Stud* **44**: 377–391.

Hansen AA, Herbert RA, Mikkelsen K, Jensen LL, Kristoffersen T, Tiedje JM *et al.* (2007). Viability, diversity and composition of the bacterial community in a high Arctic permafrost soil from Spitsbergen, Northern Norway. *Environ Microbiol* **9**: 2870–2884.

Herbold CW, Pelikan C, Kuzyk O, Hausmann B, Angel R, Berry D *et al.* (2015). A flexible and economical barcoding approach for highly multiplexed amplicon sequencing of diverse target genes. *Front Microbiol* **6**: 731.

Ishii S, Yamamoto M, Kikuchi M, Oshima K, Hattori M, Otsuka S *et al.* (2009). Microbial populations responsive to denitrification-inducing conditions in rice paddy soil, as revealed by comparative 16S rRNA gene analysis. *Appl Environ Microbiol* **75**: 7070–7078.

Jeglum JK, Rothwell RL, Berry GJ, Smith GKM. (1992). A peat sampler for rapid survey. *Can For Serv* **13**: 921–932.

Küsel K, Blöthe M, Schulz D, Reiche M, Drake HL. (2008). Microbial reduction of iron and porewater biogeochemistry in acidic peatlands. *Biogeosciences* **5**: 1537–1549.

Lee Y-J, Romanek CS, Wiegel J. (2009). *Desulfosporosinus youngiae* sp. nov., a spore-forming, sulfate-

reducing bacterium isolated from a constructed wetland treating acid mine drainage. *Int J Syst Evol Microbiol* **59**: 2743–2746.

Leininger S, Urich T, Schloter M, Schwark L, Qi J, Nicol GW *et al.* (2006). Archaea predominate among ammonia-oxidizing prokaryotes in soils. *Nature* **442**: 806–809.

Leinonen R, Sugawara H, Shumway M. (2011). The sequence read archive. *Nucleic Acids Res* **39**: D19–D21.

Loy A, Küsel K, Lehner A, Drake HL, Wagner M. (2004). Microarray and functional gene analyses of sulfate-reducing prokaryotes in low-sulfate, acidic fens reveal cooccurrence of recognized genera and novel lineages. *Appl Environ Microbiol* **70**: 6998–7009.

Ludwig W, Strunk O, Westram R, Richter L, Meier H, Yadhukumar A *et al.* (2004). ARB: a software environment for sequence data. *Nucleic Acids Res* **32**: 1363–1371.

Lüdecke C, Reiche M, Eusterhues K, Nietzsche S, Küsel K. (2010). Acid-tolerant microaerophilic Fe(II)-oxidizing bacteria promote Fe(III)-accumulation in a fen. *Environ Microbiol* **12**: 2814–2825.

Magoč T, Salzberg SL. (2011). FLASH: fast length adjustment of short reads to improve genome assemblies. *Bioinformatics* **27**: 2957–2963.

Martin M. (2011). Cutadapt removes adapter sequences from high-throughput sequencing reads. *EMBnet* **17**: 10–12.

Matzner E. (2004). Biogeochemistry of forested catchments in a changing environment: a German case study. Springer Berlin Heidelberg.

McMurdie PJ, Holmes S. (2013). phyloseq: an R package for reproducible interactive analysis and graphics of microbiome census data. *PLoS One* **8**: e61217.

McMurdie PJ, Holmes S. (2014). Waste not, want not: why rarefying microbiome data is inadmissible. *PLoS Comput Biol* **10**: e1003531.

Müller AL, Kjeldsen KU, Rattei T, Pester M, Loy A. (2015). Phylogenetic and environmental diversity of DsrAB-type dissimilatory (bi)sulfite reductases. *ISME J* **9**: 1152–1165.

Paul S, Küsel K, Alewell C. (2006). Reduction processes in forest wetlands: tracking down heterogeneity of source/sink functions with a combination of methods. *Soil Biol Biochem* **38**: 1028–1039.

Pester M, Bittner N, Deevong P, Wagner M, Loy A. (2010). A 'rare biosphere' microorganism contributes to sulfate reduction in a peatland. *ISME J* **4**: 1591–1602.

Pester M, Brambilla E, Alazard D, Rattei T, Weinmaier T, Han J *et al.* (2012a). Complete genome sequences of *Desulfosporosinus orientis* DSM765^T, *Desulfosporosinus youngiae* DSM17734^T, *Desulfosporosinus meridiei* DSM13257^T, and *Desulfosporosinus acidiphilus* DSM22704^T. *J Bacteriol* **194**: 6300–6301.

Pester M, Knorr K-H, Friedrich MW, Wagner M, Loy A. (2012b). Sulfate-reducing microorganisms in wetlands – fameless actors in carbon cycling and climate change. *Front Microbiol* **3**: 72.

Quast C, Pruesse E, Yilmaz P, Gerken J, Schweer T, Yarza P *et al.* (2013). The SILVA ribosomal RNA gene

database project: improved data processing and web-based tools. *Nucleic Acids Res* **41**: D590–D596.

R Core Team. (2015). R: a language and environment for statistical computing. R Foundation for Statistical Computing: Vienna, Austria. http://www.r-project.org/.

Reiche M, Hädrich A, Lischeid G, Küsel K. (2009). Impact of manipulated drought and heavy rainfall events on peat mineralization processes and source-sink functions of an acidic fen. *J Geophys Res Biogeosciences* **114**: G02021.

Robinson MD, McCarthy DJ, Smyth GK. (2010). edgeR: a Bioconductor package for differential expression analysis of digital gene expression data. *Bioinformatics* **26**: 139–140.

Salter SJ, Cox MJ, Turek EM, Calus ST, Cookson WO, Moffatt MF *et al.* (2014). Reagent and laboratory contamination can critically impact sequence-based microbiome analyses. *BMC Biol* **12**: 87.

Sambrook J, Russell DW. (2001). Molecular Cloning. 3rd ed. Cold Spring Harbor Laboratory Press.

Schink B, Stams AJM. (2006). Syntrophism among prokaryotes. In: Dworkin M, Falkow S, Rosenberg E, Schleifer K-H, Stackebrandt E (eds). *The Prokaryotes: Ecophysiology and Biochemistry*. Springer NY, pp 309–335.

Schmalenberger A, Drake HL, Küsel K. (2007). High unique diversity of sulfate-reducing prokaryotes characterized in a depth gradient in an acidic fen. *Environ Microbiol* **9**: 1317–1328.

Sobek JM, Charba JF, Foust WN. (1966). Endogenous metabolism of *Azotobacter agilis*. *J Bacteriol* **92**: 687–695.

Stamatakis A. (2014). RAxML version 8: a tool for phylogenetic analysis and post-analysis of large phylogenies. *Bioinformatics* **30**: 1312–1313.

Steger D, Wentrup C, Braunegger C, Deevong P, Hofer M, Richter A *et al.* (2011). Microorganisms with novel dissimilatory (bi)sulfite reductase genes are widespread and part of the core microbiota in low-sulfate peatlands. *Appl Environ Microbiol* **77**: 1231–1242.

Stoddard SF, Smith BJ, Hein R, Roller BRK, Schmidt TM. (2015). *rrn*DB: improved tools for interpreting rRNA gene abundance in bacteria and archaea and a new foundation for future development. *Nucleic Acids Res* **43**: D593–D598.

Sukenik A, Kaplan-Levy RN, Welch JM, Post AF. (2012). Massive multiplication of genome and ribosomes in dormant cells (akinetes) of *Aphanizomenon ovalisporum* (Cyanobacteria). ISME J **6**: 670–679.

Wickham H. (2009). ggplot2: elegant graphics for data analysis. Springer NY.

Wickham H. (2011). The split-apply-combine strategy for data analysis. *J Stat Softw* **40**: 1.

Yang S, Wen X, Jin H, Wu Q. (2012). Pyrosequencing investigation into the bacterial community in permafrost soils along the China-Russia crude oil pipeline (CRCOP). *PLoS One* **7**: e52730.

Yarza P, Yilmaz P, Pruesse E, Glöckner FO, Ludwig W, Schleifer K-H *et al.* (2014). Uniting the classification of cultured and uncultured bacteria and archaea using 16S rRNA gene sequences. *Nat Rev Microbiol* **12**: 635–645.
